# Robust Demodulation of eLoran Tri-State PPM via Multi-Scale Spatiotemporal Modeling

**DOI:** 10.3390/s26196035

**Published:** 2026-09-23

**Authors:** Zhe Li, Shifeng Li, Jiangbin Yuan, Ruoshui Leng, Xiangyi Wang

**Affiliations:** 1 National Time Service Center, Chinese Academy of Sciences, Xi’an 710600, China; lizhe@ntsc.ac.cn (Z.L.); yuanjiangbin@ntsc.ac.cn (J.Y.); lengruoshui24@mails.ucas.ac.cn (R.L.); wangxiangyi24@mails.ucas.ac.cn (X.W.); 2 University of Chinese Academy of Sciences, Beijing 100039, China

**Keywords:** eLoran, tri-state pulse position modulation, multi-scale spatiotemporal network, nominal-center sampling, continuous wave interference, skywave interference, cross-station interference

## Abstract

Enhanced Loran (eLoran) provides a terrestrial complement to Global Navigation Satellite Systems (GNSSs), but tri-state pulse position modulation (PPM) demodulation remains difficult under composite interference. This study proposes a label-independent Nominal-Center (NC) sampling strategy and a Multi-Scale Spatiotemporal Network (MSST-Net). NC extracts each pulse around its predicted nominal timing anchor, thereby avoiding target-dependent actual-center alignment while preserving the displacement that encodes the PPM state. MSST-Net combines multi-scale one-dimensional convolutions, Squeeze-and-Excitation channel recalibration, bidirectional long short-term memory, temporal attention, and a distance-aware mixed ordinal loss. Under a predefined controlled simulation protocol with a group- and frame-disjoint partition, the seed-42 model was evaluated on 61,008 held-out channel-realized windows generated from 372 test pulse identities belonging to 62 pulse groups across 164 nominal SNR–Stage configurations. It achieved 93.71% accuracy, 93.85% balanced accuracy, and 93.81% macro-F1. Under a separate parameter-matched cross-entropy protocol, MSST-Net (CE) achieved 92.56% accuracy, versus 91.23% for the Transformer, 89.34% for CNN-BiLSTM, and 88.29% for ResNet1D. A label-free diagnostic on 1440 receiver-recorded windows indicated a remaining simulation-to-real gap; without independently verified tri-state labels, no real-waveform accuracy was claimed. Thus, the supervised results apply to the controlled simulated benchmark and do not establish operational receiver accuracy.

## 1. Introduction

The enhanced Loran (eLoran) system operates in the 90–110 kHz band as a ground-based long-range radio navigation system [1]. Compared with satellite navigation systems, eLoran offers higher transmission power, longer propagation distances, stronger signal penetration, and greater interference resistance, making it an important terrestrial backup and complement to Global Navigation Satellite Systems (GNSSs) [2]. GNSS receivers must process very weak satellite signals and are therefore vulnerable to unintentional interference, intentional jamming, and spoofing. These threats may cause loss of service or generate misleading positioning and timing solutions [3]. Such vulnerabilities strengthen the motivation for an independent terrestrial complement such as eLoran, particularly for improving the resilience and diversity of PNT infrastructure. Beyond navigation and timing, eLoran also supports data links, such as the Eurofix data link, which transmits timing information within adjacent pulse groups using pulse position modulation (PPM). With the continuous development of high-precision terrestrial timing systems in China, eLoran data links are increasingly important for wide-area timing, differential enhancement, and synchronization of critical infrastructures. For eLoran receivers, reliable data demodulation is essential for autonomous timing, differential correction, and integrity monitoring. Accurate single-pulse state recognition, namely discriminating Early, On-time, and Late microsecond-level shifts, forms the basis of PPM demodulation and timing computation, and its classification accuracy directly determines the timing performance and data communication reliability of eLoran systems.

In eLoran signal propagation environments, interference is extremely complex. Received signals are affected not only by additive white Gaussian noise (AWGN), but also by skywave interference caused by ionospheric reflection, continuous wave interference (CWI) from co- or adjacent-frequency sources, and cross-station interference (XCI) resulting from overlapping signals from other stations or different GRI links. Existing eLoran data demodulation methods still experience performance bottlenecks under complex multi-source interference conditions, which limits the further development of eLoran systems in high-reliability PNT scenarios.

Existing eLoran data demodulation methods can be divided into three categories. The first category comprises traditional digital signal processing methods based on pulse envelope, phase, or correlation detection. The earliest cross-correlation methods [4,5] calculate the correlation peak between the received signal and a reference signal to perform PPM pulse detection and time-shift estimation. These methods are suitable for signal recognition under purely AWGN conditions but exhibit poor adaptability to complex interference. Subsequently, envelope correlation–phase detection (EC–PD) methods [6] were developed, extracting pulse envelope correlation features and carrier phase information to enable PPM state decisions at low signal-to-noise ratios. Phase spectrum smoothing demodulation (PSSD) methods [7] further apply smoothing to the phase spectrum to enhance phase discrimination under noisy conditions. Overall, these methods have clear physical interpretations and low implementation complexity, but they are generally designed for AWGN or simple noise types and do not adequately consider structural interference such as skywave, narrowband, or cross-station interference. When multiple types of interference coexist, pulse envelopes and phase structures are distorted, reducing the robustness of these methods in complex electromagnetic environments.

The second category comprises machine-learning-based data-driven demodulation methods. For example, Reference [8] proposes a multiclass support vector machine (MSVM) for eLoran demodulation. The method constructs handcrafted envelope-, phase-, and frequency-domain features and applies a linear-kernel classifier to distinguish among different PPM states. Compared with traditional envelope–phase demodulation methods, this data-driven formulation can improve classification performance under complex noise conditions by learning decision boundaries in the selected feature space. However, its dependence on handcrafted features and a linear decision function may limit its ability to represent the nonlinear waveform variations produced by composite interference.

In recent years, deep learning has been widely applied to modulation classification [9,10] and has also begun to be explored for eLoran signal demodulation and message recognition [11,12]. Reference [11] proposes an MLP-based demodulation method for the ninth eLoran pulse, which extracts 1600 key points from the received frame and constructs a lightweight fully connected network to perform end-to-end classification of 32 modulation states, demonstrating superior noise robustness compared with traditional EPD methods at low SNR. Reference [12] further proposes a GTCN-Transformer-based pulse-group message recognition algorithm, using cepstral analysis to extract pulse-group features and combining a gated temporal convolutional network with a Transformer encoder to model local temporal features and long-range dependencies, achieving good recognition performance under complex conditions including skywave, cross-station interference, and noise. Nevertheless, existing deep learning methods still have limitations: the ninth-pulse MLP method is tailored for 32-state modulation, which differs from three-state PPM in modulation structure and classification targets, and the GTCN-Transformer method focuses on pulse-group-level message recognition and relies on preprocessing steps such as cepstral analysis, which may weaken fine-grained time-domain information including pulse edges, local carrier structures, and microsecond-level shifts. Therefore, this study investigates eLoran single-pulse three-state PPM classification under a predefined controlled composite-interference model. The adopted simulation protocol enables reproducible analysis of waveform distortion and classification behavior, but it does not reproduce all synchronization, propagation, and receiver-front-end uncertainties encountered in operational eLoran systems.

Residual networks [13] and Transformer encoders [14] provide representative convolutional and self-attention-based architectures, respectively. We adapted both to the same 160-sample input and three-state output as MSST-Net, enabling task-matched evaluation without directly comparing published results obtained from different datasets or demodulation tasks.

To address these challenges, this paper proposes a single-pulse three-state PPM classification method for eLoran under complex environments. To mitigate implicit alignment issues during sample construction, a Nominal-Center (NC) alignment strategy is introduced, in which pulse samples are cropped around theoretical modulation centers rather than actual modulation centers, preventing the leakage of target-dependent alignment information and absolute sample positions. To fully exploit multi-scale features of pulse signals, a Multi-Scale Spatiotemporal Network (MSST-Net) is designed, integrating multi-scale one-dimensional convolutions, Squeeze-and-Excitation (SE) channel recalibration, bidirectional long short-term memory (BiLSTM), and temporal attention mechanisms. This architecture jointly models microsecond-level PPM shifts, local carrier structures, pulse-envelope morphology, and temporal dependencies in an end-to-end manner. Its performance is evaluated under the controlled composite-interference simulation protocol defined in Section 2.5.

The main contributions of this work are summarized as follows:

(1) A controlled eLoran signal-generation model is formulated by combining groundwave, AWGN, CWI, sparse delayed-replica skywave components, and a simplified delayed-copy overlap surrogate for XCI, thereby defining a reproducible benchmark for single-pulse three-state PPM classification;
(2) A label-independent Nominal-Center windowing strategy is formulated to retain the class-defining ±1 μs displacement relative to a common nominal timing reference and to avoid using the ground-truth PPM-shifted center during sample construction;
(3) The MSST-Net architecture is designed to fuse multi-scale spatial and temporal features, combining physical feature extraction across multiple receptive fields with sequential context modeling, significantly enhancing feature representation and robust classification under extreme interference;
(4) A mixed ordinal loss function with physical distance awareness is introduced, explicitly incorporating the ordinal relationships and distance constraints of three-state PPM into model optimization.

The scope of this study is algorithm development and controlled validation under a predefined simulated eLoran interference model. All supervised quantitative performance values reported in this paper were obtained from simulated datasets. To supplement the controlled simulation experiments, an exploratory label-free case study using archived receiver-recorded eLoran waveforms is also presented in Section 3.5. This case study examines whether the frozen simulation-trained model produces non-degenerate outputs when directly exposed to real receiver signals and identifies the remaining simulation-to-real domain gap.

The remainder of the paper is organized as follows: Section 2 presents the eLoran signal model, interference model, and the proposed method; Section 3 reports the experimental setup and results; Section 4 discusses the experimental findings; Section 5 concludes the paper.

## 2. Materials and Methods

### 2.1. eLoran Signal Model and PPM Modulation

As a long-range, high-precision, large-scale terrestrial low-frequency radio navigation and timing system, eLoran transmits signals in pulse groups [2,15,16]. Each master station (M) emits nine pulses per group, while each secondary station (S) emits eight pulses per group. Pulses within a group are transmitted at fixed instants and follow a predefined A/B phase coding sequence across successive group repetition intervals (GRIs), as shown in Figure 1, to ensure distinguishability among multiple stations and to reduce mutual interference.

The waveform of the p-th eLoran pulse is represented using a global continuous-time coordinate t. Let t denote continuous time within the pulse-group reference frame, $\tau_{p}$ the actual emission instant of the p-th pulse, and $u_{p}=t-\tau_{p}$ the local time relative to that emission instant. Furthermore, $f_{c}=100$ kHz denotes the eLoran carrier frequency, and $\sigma_{p}\in \left\{+1,-1\right\}$ denotes the A/B phase-coding symbol of the p-th pulse. With these definitions, the pulse waveform can be written as follows:

(1) $$s_{p}\left(t\right)=\left\{\begin{array}{ll} \sigma_{p}A_{0}u_{p}^{2}exp(-2u_{p}/\tau_{e})sin(2\pi f_{c}u_{p}), & 0\leq u_{p}\leq T_{pulse},\\ 0, & otherwise. \end{array}\right.$$

In Equation (1), $s_{p}\left(t\right)$ denotes the time-domain waveform of the  p-th pulse, $A_{0}$ is the amplitude coefficient, $\tau_{e}$ is the envelope time constant, and $T_{\mathrm{pulse}}$ is the effective pulse duration.

If a pulse group contains P pulses, the group signal $s_{g}(t)$ is given by

(2) $$s_{g}(t)=\sum_{p=1}^{P}s_{p}(t)$$

For a continuous transmission sequence composed of  G pulse groups, the transmitted signal $s_{\mathrm{tx}}(t)$ is expressed as

(3) $$s_{\mathrm{tx}}(t)=\sum_{g=1}^{G}s_{g}\left(t-(g-1)\mathrm{GRI}\right)$$

where *GRI* denotes the group repetition interval, expressed in microseconds.

eLoran data modulation is achieved by introducing microsecond-level shifts at the theoretical emission times $\tau_{p}^{\left(0\right)}$. Let $y_{p}\in \{-1,0,+1\}$ denote the modulation label; the actual emission time of the p-th pulse is then given by

(4) $$\tau_{p}=\tau_{p}^{\left(0\right)}+y_{p}\Delta$$

where Δ=1 μs is the fixed time shift increment. Typically, only pulses 3 through 8 participate in three-state modulation, while the remaining pulses remain at their theoretical positions. The six-dimensional label vector for the g-th pulse group is

(5) $$y_{g}=[y_{g,3},y_{g,4},\cdot \cdot \cdot {y_{g,i}\cdot \cdot \cdot y}_{g,8}{]}^{⊤},i\in \{3\cdot \cdot \cdot 8\},y_{g,i}\in \{-1,0,+1\}$$

This model provides the fundamental time-domain representation of eLoran single pulses and pulse groups, clarifying the physical implementation of three-state PPM along the time axis and establishing the basis for subsequent single-pulse sample construction and classification tasks.

### 2.2. Multi-Interference Channel Model

In practical propagation environments, eLoran received signals are simultaneously affected by propagation paths, continuous wave interference, cross-station interference, and thermal noise, as illustrated in Figure 2. The received signal is modeled as

(6) $$r(t)=r_{\mathrm{ground}}(t)+r_{\mathrm{sky}}(t)+i_{\mathrm{CWI}}(t)+i_{\mathrm{XCI}}(t)+n(t).$$

The groundwave component [17] is expressed as:

(7) $$r_{\mathrm{ground}}(t)=\alpha_{0}s_{\mathrm{tx}}(t-\tau_{0}),$$

where $\alpha_{0}$ and $\tau_{0}$ denote the propagation gain and propagation delay of the groundwave, respectively. The skywave component models multipath propagation [18,19]:

(8) $$r_{\mathrm{sky}}(t)=\sum_{l=1}^{L}\alpha_{l}s_{\mathrm{tx}}(t-\tau_{l}),$$

where L is the number of skywave paths, and $\alpha_{l}$ and $\tau_{l}$ denote the amplitude attenuation coefficient and propagation delay of the l-th path, respectively.

Continuous wave interference (CWI) is expressed as:

(9) $$i_{\mathrm{CWI}}(t)=\sum_{m=1}^{M}A_{m}cos(2\pi f_{m}t+\phi_{m}),$$

where M is the number of interference components, and $f_{m}$, $A_{m}$, and $\phi_{m}$ denote the frequency, amplitude, and initial phase of the  m-th component. Cross-station interference (XCI) approximates the overlapping signals from other stations along the time axis:

(10) $$i_{\mathrm{XCI}}(t)=\sum_{q=1}^{Q}\beta_{q}s_{\mathrm{tx}}(t-\delta_{q}),$$

where  Q is the number of interference copies, $\beta_{q}$ is the amplitude scaling factor, and $\delta_{q}$ is the relative time delay. Additive noise is modeled as Gaussian white noise:

(11) $$n(t)∼N(0,\sigma_{n}^{2}).$$

The skywave and XCI components are intentionally formulated as first-order parametric models for controlled algorithm development rather than as complete site-specific propagation models. The skywave term represents a sparse set of delayed and attenuated replicas of the transmitted waveform, thereby capturing the principal pulse-broadening, trailing, and multipath-superposition effects without explicitly modeling time-varying ionospheric dispersion. The XCI term uses delayed and scaled replicas of the transmitted waveform to isolate temporal-overlap distortion. It does not explicitly reproduce an interfering station’s independent Group Repetition Interval, A/B phase-coding sequence, carrier-phase offset, or transmitter-specific waveform. These simplifications make it possible to control individual interference factors and evaluate the demodulation algorithm under repeatable conditions.

The component-wise power definitions and the nominal signal-to-noise ratio convention used for dataset generation are specified in Section 2.5.1.

### 2.3. Problem Definition and Nominal-Center Sampling

#### 2.3.1. Problem Definition

The core task of this study is to perform three-state classification of single pulses within the received sequence under complex interference. Let $x_{g,p}\in R^{N}$ denote the extracted sample of the  p-th pulse in the g-th group from the received signal, and let $y_{g,p}\in \{-1,0,+1\}$ represent the corresponding label, indicating Early, On-time, and Late PPM states, respectively. The objective is to learn a nonlinear mapping function

(12) $$f_{\theta }:x_{g,p}\mapsto {\hat{y}}_{g,p},\;{\hat{y}}_{g,p}\in \{-1,0,+1\},$$

where θ represents the model parameters. Given a sample set $D=\{(x_{g,p},y_{g,p})\}$, the training goal is to minimize the expected risk

(13) $$\underset{\theta }{min}\;E_{(x,y)∼D}\left[l(f_{\theta }(x),y)\right],$$

where l(⋅,⋅) denotes a classification loss function.

To ensure that the dataset strictly reflects the actual eLoran transmission mechanism, a protocol-constrained top-down generation process is designed. The process consists of three stages: first, a complete 210-bit data frame is generated at the message level according to the standard specification; second, the frame is divided into 30 pulse groups according to the protocol structure, with each group carrying seven bits of information; and finally, each group’s bit sequence is mapped to the corresponding pulse modulation pattern according to the modulation mapping table. This hierarchical procedure preserves the protocol-specified temporal features, phase coding rules, and intra-group structural consistency at the sample level. The overall process is illustrated in Figure 3.

#### 2.3.2. Nominal-Center Single-Pulse Sample Construction Method

In each eLoran pulse group, pulses 3–8 carry tri-state PPM information. Let $y_{g,p}\in \left\{-1,0,+1\right\}$ denote the state of pulse p in group g, and let Δ=1 μs denote the elementary PPM displacement. A receiver first establishes a group-level local time reference $t_{g}^{ref}$, for example, from the non-data pulse and the receiver tracking loop. Based on the known inter-pulse spacing $d_{p}^{0}$, the nominal timing anchor of the p-th data pulse is

(14) $$t_{g,p}^{0}=t_{g}^{ref}+d_{p}^{0},t_{g}^{ref}=\left(g-1\right)GRI+\tau_{0.}$$

The actual arrival time of the PPM-shifted pulse is ${\;t}_{g,p}^{act}=t_{g,p}^{0}+y_{g,p}\Delta .$ Let the estimated nominal timing anchor be $\;{\hat{t}}_{g,p}^{0}=t_{g,p}^{0}+\varepsilon_{sync,g\;}$, where $\varepsilon_{sync,g}$ represents a possible group-level timing-reference error. The residual pulse displacement relative to the extraction anchor is therefore

(15) $$t_{g,p}^{act}-{\hat{t}}_{g,p}^{0}=y_{g,p}\Delta -\varepsilon_{sync,g}.$$

The nominal-center (NC) strategy centers the extraction window on ${\hat{t}}_{g,p}^{0}$, rather than on the unknown PPM-shifted arrival time. Consequently, the sample construction procedure is independent of ${\;y}_{g,p}$, while the class-dependent temporal displacement is retained within the window. In the primary simulated dataset used in this study, $\varepsilon_{sync,g}=0$; therefore, the main results do not quantify robustness to nonzero synchronization errors.

For a sampling rate $f_{s\;}$, the nominal anchor index is $n_{g,p}^{0}=\mathrm{round}\left({\hat{t}}_{g,p}^{0}f_{s}\right).\;$ The extracted discrete-time pulse window is defined as

(16) $$x_{g,p}\left[k\right]=r\left[n_{g,p}^{0}-N_{L}+k\right],k=0,\ldots ,N_{L}+N_{R}-1.$$

In formal experiments, $f_{s}=2\;MHz$ and $N_{L}=N_{R}=80$, giving a 160-sample $\left(80\;\mu s\right)$ window. Since a 1 μs displacement corresponds to two samples, the Early, On-time, and Late states occur at offsets of −2, 0, and +2 samples, respectively, when $\varepsilon_{sync,g}=0$. The filtering and other signal-generation settings are provided in Section 2.5.

For the controlled comparison in Section 3.4.1, three window-center definitions are considered. Nominal-Center (NC) uses the same common nominal timing anchor for all three classes and does not require the unknown PPM state. Actual-Center (AC) is an oracle diagnostic that uses the ground-truth PPM-shifted arrival position and is therefore unavailable during inference. The third comparator is a signal-driven Peak-Lock (PL) rule that searches within ±8 μs of the nominal anchor and selects the sample with the maximum absolute received amplitude.

### 2.4. MSST-Net Architecture

To address the challenges of microsecond-level shift ambiguity, waveform distortion, and insufficient modeling of temporal dependencies in single-pulse three-state PPM recognition under complex interference, this paper proposes a Multi-Scale Spatiotemporal Network (MSST-Net). The network first employs multi-scale convolutions at the front end to extract local features at different temporal resolutions, combined with Squeeze-and-Excitation (SE) channel recalibration. A two-layer bidirectional long short-term memory (BiLSTM) network with an attention mechanism is then used to model temporal dependencies within each pulse. Finally, a mixed ordinal loss with physical distance awareness is applied to perform three-state PPM classification. The overall architecture of MSST-Net is illustrated in Figure 4.

#### 2.4.1. Input Normalization

To reduce the influence of amplitude scale differences among samples, each single pulse is first normalized. Let the original input pulse be $p\in R^{T\times 1}$ with T=160. The normalized result is:

(17) $$x_{\mathrm{in}}=\frac{p-\mu_{p}}{\sqrt{\sigma_{p}^{2}+\eta }},$$

where $\mu_{p}$ and $\sigma_{p}$ denote the mean and standard deviation of the sample, respectively, and $\eta =10^{-8}$ is a small constant for numerical stability. This processing suppresses amplitude bias and dynamic range differences, allowing the network to focus on relative waveform structures rather than absolute amplitudes.

#### 2.4.2. Multi-Scale Convolution Feature Extraction and Channel Recalibration

Discriminative information in eLoran single pulses exhibits significant multi-temporal-scale characteristics: small PPM shifts appear as subtle timing deviations near the pulse leading edge; local carrier oscillations reflect medium-scale periodic structures; pulse envelope broadening, distortion, and trailing occur over larger receptive fields. A parallel multi-branch one-dimensional convolutional structure is designed to jointly model local features at different scales. Let the input feature be x, and the output of the k-th convolutional branch is

(18) $$F_{k}=B_{k}(x),\;k\in \{1,2,3\},$$

where the design of the three branches is illustrated in Figure 5.

- Micro-shift perception branch: convolution kernel $K_{1}=5$, used to capture subtle timing deviations near the pulse leading edge, covering the typical shift range of three-state PPM.
- Local carrier extraction branch: convolution kernel K2 = 21. At the 2 MHz sampling rate, this kernel spans approximately 10.5 μs, corresponding to approximately one carrier cycle at fc = 100 kHz.
- Envelope morphology modeling branch: convolution kernel K3 = 15 and a dilation factor of 5, yielding an effective receptive field of approximately 35.5 μs to capture large-scale variations such as pulse-envelope broadening, distortion, and trailing.

The outputs of these branches are concatenated along the channel dimension to obtain fused features:

(19) $$F_{\mathrm{ms}}=Concat(F_{1},F_{2},F_{3}).$$

After multi-scale convolution fusion, Squeeze-and-Excitation (SE) channel recalibration [20] is applied. For $F_{\mathrm{ms}}$, a global average pooling (GAP) generates a channel-wise statistic vector, which is mapped through two fully connected layers to produce channel weights:

(20) $$s=\mathrm{Sigmoid}\left(W_{2}\;\phi (W_{1}\;\mathrm{GAP}(F_{\mathrm{ms}}))\right)$$

where ϕ(⋅) is the ReLU activation function. The recalibrated features are:

(21) $${\tilde{F}}_{\mathrm{ms}}=s⊙F_{\mathrm{ms}}.$$

where ⊙ denotes element-wise multiplication along channels. The basic feature-extraction unit combines multi-scale convolution, SE recalibration, and pooling and is stacked in three stages. Feature channels increase progressively from 1→32→64→128, while the temporal dimension is reduced from 160→80→40→16. The temporal dimension changes from 160 to 80 after the first MaxPool1d(2), from 80 to 40 after the second MaxPool1d(2), and from 40 to 16 after AdaptiveAvgPool1d(16).

#### 2.4.3. Two-Layer BiLSTM Temporal Modeling and Attention Aggregation

After the three-stage convolution and pooling, the feature tensor is reshaped into a temporal sequence of length $T^{\prime }=16$, with feature dimension 128 per time step: $\{x_{t}{\}}_{t=1}^{T^{\prime }}$. A two-layer bidirectional long short-term memory network (BiLSTM) [21,22] is used to model temporal dependencies. The hidden state at time t is

(22) $$h_{t}=\left[\vec{\mathrm{LSTM}}(x_{t},{\vec{h}}_{t-1})\;∥\;\overset{\leftarrow }{\mathrm{LSTM}}(x_{t},{\overset{\leftarrow }{h}}_{t+1})\right].$$

Each direction contains 64 hidden units, yielding an output dimension of 128 after concatenation. An MLP-based attention mechanism [23,24] is applied to the BiLSTM output sequence $\{h_{t}{\}}_{t=1}^{T^{\prime }}$, assigning different weights to emphasize discriminative temporal regions. The attention score is

(23) $$e_{t}=u_{a}^{⊤}tanh(W_{a}h_{t}+b_{a})$$

The scores are normalized to obtain the attention weights:

(24) $$\alpha_{t}=\frac{exp(e_{t})}{\sum_{i=1}^{T^{\prime }}exp(e_{i})},$$

The final context vector is

(25) $$c=\sum_{t=1}^{T^{\prime }}\alpha_{t}h_{t},$$

This mechanism aggregates the coarse-grained temporal regions in the pulse that are most critical for state discrimination (e.g., rising edge versus trailing segment). At $f_{s}=2$ MHz, the 160-sample input spans 80 μs. After the temporal-length transformation 160→80→40→16, the attention module operates on 16 temporal steps. The microsecond-level PPM shift information is first extracted and encoded by the front-end convolutional layers before attention-based temporal aggregation. The attention weights are illustrated in Figure 6.

#### 2.4.4. Output Layer and Mixed Ordinal Loss with Physical Distance Awareness

After obtaining the context vector c, a dropout layer is applied to mitigate overfitting [25], followed by a fully connected layer to output logits for the three PPM states:

(26) z=FC(Dropout(c))

The three PPM states possess an inherent ordinal structure and are assigned the physical labels $s_{-1}=-1$, $s_{0}=0$, and ${\;s}_{+1}=+1$, corresponding to the Early, On-time, and Late states, respectively. Using standard cross-entropy loss alone treats the three classes as independent discrete categories, ignoring the ordinal relationship among the three PPM states. To address this, a physical distance-aware term is introduced. Let the softmax output probabilities be $p_{-1}\;,p_{0},\;\mathrm{and}\;p_{+1}$, and define the soft physical state estimate as

(27) $${\hat{y}}_{soft}=\sum_{k\in \left\{-1,0,+1\right\}}k\;p_{k}=\left(-1\right)p_{-1}+0\cdot p_{0}+\left(+1\right)p_{+1},$$

and the distance penalty term as

(28) $$L_{dist}={\left(\hat{{\hat{y}}_{soft}}-s_{y}\right)}^{2},$$

The distance term assigns a larger penalty to predictions that are farther from the ground-truth state in the physical label space. Consequently, a direct Early↔Late confusion receives a larger penalty than an adjacent Early↔On-time or On-time↔Late confusion.

The overall loss function is

(29) $$L_{mixed}=L_{\mathrm{CE}}+\lambda L_{\mathrm{dist}},\lambda =0.5$$

where  λ=0.5. This mixed loss preserves discrete classification capability while incorporating the physical proximity of the three-state labels into the optimization process [26], thereby helping to suppress cross-level misclassification.

Cross-entropy preserves the categorical discrimination capability of the model, whereas the distance-aware term regularizes the physical ordering of the three PPM states. The mixed loss is therefore designed to reduce physically inconsistent cross-level predictions without replacing the conventional classification objective. Its empirical contribution is evaluated independently in Section 3.4.3.

In summary, MSST-Net constructs a complete processing pipeline: sample normalization, multi-scale convolutional local modeling, SE channel recalibration, BiLSTM temporal dependency modeling, attention aggregation, and physical distance-aware classification. The network captures microsecond-level shifts, local carrier details, and overall envelope evolution, providing a unified modeling framework for robust single-pulse three-state recognition of eLoran signals under complex interference.

### 2.5. Experimental Configuration

This section specifies the simulated channel conditions, nominal power conventions, dataset construction and partitioning protocol, model training and evaluation settings, and the exploratory protocol applied to receiver-recorded waveforms.

#### 2.5.1. Channel and Interference Configuration

All simulated waveform components were generated at a sampling frequency of $f_{s}=2\;MHz$ and an eLoran carrier frequency of $f_{c}=100\;kHz$. As summarized in Table 1, the channel conditions were organized into four cumulative interference stages: Stage 1 included AWGN only; Stage 2 added CWI; Stage 3 additionally included skywave propagation; and Stage 4 further incorporated XCI.

For dataset generation, the continuous-time model in Equation (6) was implemented in discrete time. Before band-pass filtering, the composite received sequence was

(30) $$r_{\left(pre\right)}\left[n\right]=s_{g}\left[n\right]+s_{\left(sky\right)}\left[n\right]+i_{\left(CWI\right)}\left[n\right]+i_{\left(XCI\right)}\left[n\right]+w\left[n\right]$$

where $s_{g}\left[n\right]$, $s_{sky}\left[n\right]$, $i_{CWI}\left[n\right]$, $i_{XCI}\left[n\right]$, and $w\left[n\right]$ denote the groundwave, skywave, CWI, XCI, and AWGN components, respectively.

For any N-sample sequence $x\left[n\right]$, the sequence-averaged power was defined as

(31) $$P_{x}=1/N\sum_{\left(n=0\right)}^{\left(N-1\right)}x^{2}\left[n\right]$$

All component powers were computed over the complete synthesized sequence before band-pass filtering, rather than over the subsequently extracted 160-sample pulse windows.

The nominal pre-filter signal-to-noise ratio was swept from −20 to 20 dB in 1 dB increments. It was defined as the sequence-level groundwave-to-AWGN ratio:

(32) $$SNR_{\left(nom\right)}=10log_{10}\left(P_{g}/P_{w}\right),P_{w}=P_{g}10^{\left(-SNR_{\left(nom\right)}/10\right)}$$

where $P_{g}$ and ${\;P}_{w}$ denote the sequence-averaged groundwave and AWGN powers, respectively. For the generated zero-mean AWGN, $P_{w}$ corresponds to the noise variance.

At ${SNR}_{nom}=-20\;dB$, Equation (32) gives $P_{w}=100P_{g}$. Because eLoran is a pulsed signal, the sequence-level power $\;P_{g}$ includes the inter-pulse intervals and is therefore lower than the average groundwave power within an active pulse window. Consequently, ${SNR}_{nom}$ should not be interpreted as the local SNR of an extracted pulse.

Four candidate CWI components were used, with the interference-frequency/SIR pairs given by $\left(97.2222\;kHz,\;-7.00\;dB\right)$, $\left(101.3333\;kHz,\;-10.00\;dB\right)$, $\left(103.6666\;kHz,\;-9.21\;dB\right)$, and $\left(99.1111\;kHz,\;-8.24\;dB\right)$. Here, each SIR was referenced to the sequence-averaged groundwave power $P_{g}$ defined in Equation (32). CWI configuration $K\in \left\{1,2,3,4\right\}$ activated the first K components in the listed order.

The aggregate CWI sequence and the amplitude of each component were generated as

(33) $$i_{\left(CWI\right)}\left[n\right]=\sum_{\left(k=1\right)}^{K}A_{k}cos\left(2\pi f_{k}n/f_{s}+\phi_{k}\right),\;P_{\left(CWI,k\right)}=P_{g}10^{\left(-SIR_{k}/10\right)},A_{k}=\sqrt{\left(2P_{\left(CWI,k\right)}\right)}$$

where $f_{k}$, $A_{k}$, and $\phi_{k\;}$ denote the frequency, amplitude, and initial phase of the k-th CWI component, respectively.

For Stages 2–4, the CWI configuration K was selected using the fixed-seed generation procedure and stored in the dataset manifest. Consequently, the selected CWI components were exactly reproducible for every generated sequence.

The groundwave propagation delay was fixed at $\tau_{0}=30\;\mu s$. Two candidate skywave paths were defined by the excess-delay/amplitude-ratio pairs $\left(37\;\mu s,-6\;dB\right)\;$ and $\left(53\;\mu s,-9\;dB\right)$. Thus, their total delays relative to the transmitted waveform were 67 and 83 μs, respectively. Depending on the fixed-seed configuration, either one or both paths were activated in Stages 3 and 4.

(34) $$s_{\left(sky\right)}\left[n\right]=\sum_{\left(l=1\right)}^{L}\alpha_{l}s_{\left(tx\right)}\left[n-d_{0}-d_{l}\right],\alpha_{l}=10^{\left(G_{l}/20\right)}$$

where $d_{0}$ is the discrete groundwave propagation delay, $d_{l}$ is the excess delay of the  l-th skywave path, and $G_{l}$ is its amplitude ratio in decibels.

In Stage 4, XCI was generated using Q=2 and scaled replicas of the transmitted sequence:

(35) $$\begin{array}{c} i_{XCI}\left[n\right]=\sum_{q=1}^{Q}\beta_{q}s_{tx}\left[n-d_{q}\right],Q=2,\\ \beta_{q}∼U\left(0.1,0.3\right),\\ \delta_{q}∼U\left(1,8000\right)\;\mu s,{\;\;d}_{q}=\mathrm{round}\left(\delta_{q}f_{s}\right). \end{array}$$

The amplitude factors and relative delays were sampled independently for the two replicas.

In the offline experiments, AWGN was added before filtering. Each complete received sequence was then processed using a zero-phase forward–backward Butterworth band-pass filter with a design-order parameter of 6 and a passband of 95–105 kHz. Filtering was performed on the complete sequence before pulse extraction to avoid window-level boundary artifacts. The filtered sequence was subsequently segmented into 160-sample windows using the Nominal-Center strategy described in Section 2.3.2. No post-filter SNR re-estimation was performed.

Accordingly, all SNR values reported for the simulated MSST-Net and MSVM experiments refer to the nominal pre-filter, sequence-level groundwave-to-AWGN power ratio defined in Equation (32). The skywave, CWI, and XCI components are excluded from both the numerator and denominator of Equation (32). Therefore, ${SNR}_{nom}$ denotes a groundwave-to-AWGN power ratio rather than a composite signal-to-interference-plus-noise ratio.

#### 2.5.2. Dataset Construction and Manifest-Based Disjoint Partitioning

The simulated benchmark comprised 41 nominal SNR values from −20 to 20 dB and four interference stages, yielding 41×4=164 SNR–Stage configurations. A single MSST-Net model was trained using the pooled training data from all configurations. The AWGN-only evaluation used the Stage 1 test realizations, whereas the mixed-interference evaluation pooled equal-sized test realizations from Stages 1–4. Thus, these evaluations represent two test views of the same trained model rather than results from separately trained models.

Before channel realization, a deterministic seed-42 manifest partitioned 417 unique pulse groups into 292 training, 63 validation, and 62 test groups. All six PPM-modulated pulses from the same group remained in the same subset, and each group retained its split membership across all 164 configurations. The training, validation, and test group-identifier sets were pairwise disjoint, and no pulse-level random re-splitting was performed for model training, baseline evaluation, or ablation experiments. Because each group contributed six modulated pulses, the three subsets contained 1752, 378, and 372 unique pulse identities, respectively.

For protocol-oriented organization, the groups were packed into 10 training, three validation, and three test manifest frames, with at most 30 groups per frame; the final frames contained 22, three, and two groups, respectively. These split-specific manifest frames are simulation-organizational units and should not be interpreted as receiver-recorded physical frames. At the unique-identity level, the Early, On-time, and Late classes contained 782, 938, and 782 pulses, respectively. Because of the mild imbalance toward the On-time class, balanced accuracy and macro-F1 were reported together with overall accuracy.

Applying the 2502 unique pulse identities to all 164 configurations generated 410,328 channel-realized windows: 287,328 for training, 61,992 for validation, and 61,008 for testing. These windows represent repeated channel realizations of 2502 underlying pulse identities rather than 410,328 independent identities. The Stage 1 AWGN-only test view contained 372×41=15,252 windows, whereas the pooled Stage 1–4 test view contained 372×41×4=61,008 windows. Deterministic configuration-specific seeds controlled the stochastic channel parameters. As noted in Section 2.3.2, the primary dataset assumed $\varepsilon_{sync,g}=0$; therefore, the reported results do not establish robustness to nonzero synchronization-reference errors.

#### 2.5.3. Training and Evaluation Settings

The reported model was trained using AdamW [27] with an initial learning rate of $5\times 10^{-4}$, a weight decay of $1\times 10^{-4}$, a batch size of 64, and a maximum of 50 epochs. All other AdamW parameters were left at their PyTorch defaults. A ReduceLROnPlateau scheduler monitored the validation loss and reduced the learning rate by a factor of 0.5 after five epochs without improvement. Early stopping also monitored the validation loss, with a patience of 15 epochs. Gradient clipping was applied with a maximum norm of 1.0. The mixed ordinal loss used λ=0.5, and each input window was normalized independently using the procedure defined in Section 2.4.1. Model selection was based exclusively on the minimum validation loss. For the formal seed-42 run, the retained checkpoint was obtained at epoch 17, with a mixed ordinal validation loss of 0.20350.

The formal training run used Python 3.12.1 and PyTorch 2.7.1+cu118. Seed 42 was used for model initialization and the stochastic operations in the training pipeline. The main results were obtained from this single formal training run and are therefore reported as single-run estimates rather than as means and standard deviations across multiple training seeds.

An additional parameter-matched benchmark compared MSST-Net (CE) with task-adapted ResNet1D [13], CNN-BiLSTM [21,22], and Transformer [14] classifiers. ResNet1D used a seven-sample convolutional stem and six residual blocks with 32, 64, and 120 channels. CNN-BiLSTM used three convolutional layers with 32, 64, and 128 channels, followed by a two-layer bidirectional LSTM with 80 hidden units per direction and attention pooling. The Transformer used a two-layer strided convolutional tokenizer and four encoder layers with a 96-dimensional embedding, four attention heads, and a 192-dimensional feed-forward sublayer. MSST-Net (CE) retained the architecture in Section 2.4 but used cross-entropy instead of the mixed ordinal loss.

All four models used the same 160-sample Nominal-Center inputs, per-window z-score normalization, and immutable group- and manifest-frame-disjoint split. Each was trained with cross-entropy and AdamW using seed 42, a batch size of 512, an initial learning rate of 5 × 10^−4^, a weight decay of 1 × 10^−4^, and identical scheduling, checkpoint-selection, early-stopping, and evaluation procedures. No model-specific tuning was performed. The pooled test set contained 61,008 windows, including 15,252 in each stage. Trainable-parameter counts ranged from 350,787 to 359,035. Batch-1 latency was measured on the same NVIDIA GeForce RTX 4060 Laptop GPU as the mean of 100 synchronized forward passes after 20 warm-up iterations. Results are single-run estimates without across-seed confidence intervals. Cross-entropy was used for all four models to reduce confounding between architecture and loss-function design.

Applying the proposed mixed ordinal loss only to MSST-Net would prevent the observed differences from being attributed primarily to architecture. The complete mixed-ordinal-loss model is therefore reported separately.

Overall accuracy (ACC), balanced accuracy, and macro-averaged F1 score (macro-F1) were used to evaluate three-state pulse classification. Balanced accuracy was computed as the unweighted mean recall across the Early, On-time, and Late classes, whereas macro-F1 was computed as the unweighted mean of their class-wise F1 scores. Each pulse-level three-state decision was treated as one PPM symbol. The symbol error rate was therefore calculated as  SER=1−ACC.  SER was reported to facilitate comparison with previous eLoran demodulation studies [6,8].

#### 2.5.4. Protocol for the Label-Free Receiver-Recorded Waveform Case Study

In addition to the simulated benchmark, archived eLoran waveforms recorded at three receiving sites—Lintong, Wuwei, and Shanshan—were used for an exploratory label-free case study. The recordings were provided by the National Time Service Center and were not acquired by the authors. For each site, the first 6,000,000 samples of a recording with a nominal sampling rate of 1 MHz were analyzed, corresponding to 6.0 s or approximately 100 GRIs. Independently verified pulse-level tri-state labels were unavailable. Therefore, the recordings were used only for label-free diagnostic analysis and not for quantitative evaluation of receiver-recorded demodulation accuracy.

Each recording was resampled from 1 to 2 MHz using polyphase resampling. The first 20 GRIs at each site formed a calibration segment used exclusively for label-free synchronization.

Consistent with the simulated preprocessing pipeline, each input window contained 160 samples, was centered on the nominal timing anchor, and spanned ±40 μs. No target-domain fine-tuning, optimizer update, pseudo-label generation, or model-output-based synchronization or alignment was performed.

Pulses 3–8 in the 80 evaluation GRIs yielded 480 candidate pulse windows per site and 1440 windows in total. The analysis was restricted to the predicted-class distribution, effective class count, predictive confidence, and normalized predictive entropy, together with off-pulse and protocol-reference sanity controls.

## 3. Results

This section evaluates MSST-Net under AWGN-only and cumulative mixed-interference conditions, compares it with task-matched and contextual reference methods, examines its main design components through controlled ablations, and presents an exploratory label-free analysis of receiver-recorded waveforms. The evaluation structure is summarized in Table 2.

Unless otherwise stated, all simulated experiments used the fixed group-disjoint manifest described in Section 2.5.2, with no pulse-level re-splitting. For MSST-Net and MSVM, SNR denotes the nominal pre-filter, sequence-level groundwave-to-AWGN ratio defined in Section 2.5.1. Results reproduced from conventional DSP studies retain their source-specific SNR conventions and are therefore used only as contextual references.

### 3.1. Performance on the AWGN-Only Dataset

This subsection evaluates MSST-Net on the predefined AWGN-only Stage 1 test subset. Table 3 reports selected SNR points, whereas Figure 7 presents the complete SNR-conditioned ACC and SER trends.

At −20 dB, MSST-Net correctly classified 361 of 372 test pulses, yielding an ACC of 97.04% and an SER of 2.96%. At −16 dB, 371 of 372 pulses were classified correctly, and no errors occurred at SNRs of −15 dB or higher in this finite test set. The small non-monotonic variations from −20 to −18 dB reflect finite, independently generated channel realizations and do not indicate improved physical performance at lower SNR.

Figure 8 presents the confusion matrix for the complete AWGN-only test subset. The remaining errors occur mainly between adjacent PPM states, while direct Early–Late confusions are rare, consistent with the ordinal class structure.

### 3.2. Performance on the Mixed-Interference Dataset

This subsection evaluates the MSST-Net checkpoint under the predefined composite simulated channel model, which includes AWGN, CWI, skywave propagation, and XCI. The test set comprises 41 nominal SNR values and four interference stages, with 372 held-out pulses for each SNR–Stage configuration, yielding 61,008 test pulses in total. All configurations follow the same predefined group- and frame-disjoint manifest.

Table 4 and Figure 9 pool the four Stage subsets at each nominal SNR, giving 1488 test windows per SNR point. Because the nominal SNR controls only the groundwave-to-AWGN ratio and the structured-interference parameters vary across SNR–Stage configurations, the resulting curves do not represent a pure SNR sweep under a fixed composite channel. Their local non-monotonicity reflects the combined effects of AWGN and configuration-specific structured interference; overall and Stage-wise metrics therefore provide the more informative robustness summaries.

To examine performance across different levels of simulated channel complexity, Table 5 and Figure 10 stratify the fixed mixed-interference test set by Stage. The corresponding samples were selected from the predefined test manifest according to their Stage labels; the model was not retrained for individual Stages, and no test pulses were re-generated or re-drawn during Stage-wise evaluation.

Performance decreased monotonically with cumulative channel complexity. ACC fell from 99.72% in Stage 1 to 93.00% in Stage 2, a reduction of 6.72 percentage points, and then to 91.67% and 90.44% in Stages 3 and 4, respectively. Balanced accuracy showed the same trend. Under the adopted simulation protocol, the cumulative structured-interference conditions were therefore substantially more challenging than AWGN alone.

Figure 11 summarizes all 61,008 held-out simulated test windows. MSST-Net achieved an ACC of 93.71%, a balanced accuracy of 93.85%, and a macro-F1 score of 93.81%. The Early, On-time, and Late recalls were 96.08%, 91.72%, and 93.73%, respectively. Adjacent-state errors accounted for 5.88% of all windows, whereas direct Early–Late confusions accounted for only 0.41%, showing that most residual errors occurred between neighboring states under the simulated protocol.

### 3.3. Comparison with Task-Matched Baselines and Contextual References

The evaluation comprised task-matched and contextual comparisons. The task-matched evaluation included the original comparison of the complete MSST-Net with linear MSVM and a lightweight CNN, and a separate CE-controlled comparison of MSST-Net (CE), ResNet1D, CNN-BiLSTM, and Transformer. All task-matched methods used the same 160-sample Nominal-Center input and fixed group- and manifest-frame-disjoint split; the four modern architectures additionally shared the protocol in Section 2.5.3. Analytical and conventional DSP results [4,6,28] were retained only as contextual references because their signal models, SNR definitions, and evaluation conditions differed.

On the common 61,008-window test set, MSST-Net achieved an ACC of 93.71% and an SER of 6.29%, compared with 86.61%/13.39% for linear MSVM and 87.46%/12.54% for the lightweight CNN. The ACC difference between MSST-Net and MSVM increased from 0.54 percentage points in Stage 1 to 6.91, 9.37, and 11.59 percentage points in Stages 2–4, respectively. Thus, the observed difference from the linear baseline became larger as the simulated channel complexity increased. Because the lightweight CNN differs from MSST-Net in several architectural dimensions, it serves as a composite task-matched reference rather than a single-factor ablation; individual components are examined in Section 3.4.

Recent deep-learning-based eLoran studies [11,12] were not used as direct numerical baselines because their tasks and evaluation units differ from the present single-pulse three-state classification problem. Guo et al. [11] address 32-state ninth-pulse demodulation using 1600-point frame-level inputs, whereas Zhang et al. [12] perform pulse-group-level message recognition using cepstral preprocessing and a GTCN-Transformer. Adapting either method would require changing its original input, output, and training objective; the studies are therefore discussed as related work rather than plotted as task-matched baselines.

Figure 12 shows the semilogarithmic SER trends over the reported SNR labels. On the common Stage 1 test split, MSST-Net achieved SERs of 2.96% and 0.27% at nominal pre-filter SNRs of −20 and −16 dB, respectively. No errors occurred at −15 dB or higher among the 372 test pulses per SNR point. These finite-sample observations do not imply theoretical zero-error performance or equivalence to post-filter in-band SNR.

The analytical and DSP curves retain their source-specific signal representations, sampling settings, and AWGN assumptions. Consequently, their numerical differences from MSST-Net should not be interpreted as performance gains under identical receiver inputs. Table 6 reports the pooled performance and computational characteristics of the four CE-trained architectures. Their parameter counts were kept within a narrow range to reduce capacity-related confounding.

MSST-Net (CE) achieved the highest ACC, balanced accuracy, and macro-F1 among the four architectures. Its ACC exceeded those of the Transformer, CNN-BiLSTM, and ResNet1D by 1.328, 3.221, and 4.264 percentage points, respectively. Parameter counts differed by at most 2.35%, and MSST-Net was not the largest model, reducing model size as an explanation for the ranking. However, its 2.289 ms batch-1 latency exceeded the 1.078–1.833 ms range of the external baselines, indicating an accuracy–latency trade-off (see Figure 13).

All models approached ceiling performance in Stage 1 (99.521–99.764%). MSST-Net retained accuracies of 91.667%, 89.988%, and 88.808% in Stages 2–4. The largest observed reduction for every model occurred between Stages 1 and 2. Because the stage subsets were generated separately, these changes should not be interpreted as paired interference ablations. Figure 14 reports the accuracy difference $ACC_{MSST}-ACC_{baseline}$ for the pooled set and each stage.

MSST-Net achieved the highest observed accuracy in every subset. Relative to the strongest external baseline, the Transformer, its advantages were 1.639, 2.609, and 0.983 percentage points in Stages 2–4. Thus, model differences were small under Stage 1 AWGN-only conditions but became more visible in the structured-interference subsets. These single-seed results establish an observed within-protocol ranking only.

### 3.4. Ablation Study

All ablations used the same fixed split manifest, pulse identities, labels, and SNR–Stage configurations as the main evaluation. Unless explicitly identified as multi-seed experiments, the reported variants were trained once with seed 42. The analyses examine sample construction, network architecture and preprocessing, and the mixed ordinal loss.

#### 3.4.1. Ablation of the Sample-Construction Strategy

To quantify the effect of the window-center definition, we compared Nominal-Center (NC), ground-truth Actual-Center (AC), and signal-driven Peak-Lock (PL) construction. The three experiments used the same group- and frame-disjoint split manifest, the same 372 held-out pulse identities, the same 164 SNR–Stage configurations, the same MSST-Net architecture, and identical optimization and model-selection settings. Each strategy generated its own 160-sample windows for the corresponding training, validation, and test identities. Therefore, the only intended experimental difference was the rule used to determine the window center. All reported results correspond to a single fixed training seed of 42. The quantitative comparison of the three sample-construction strategies is summarized in Table 7.

NC achieved an ACC of 93.709% and a macro-F1 score of 93.812%. In contrast, AC achieved an ACC of 37.634%. Its predictions collapsed entirely to the On-time class: all 61,008 test samples were assigned to the middle state. This behavior does not indicate that ground-truth centering provides a useful label-dependent feature. Instead, recentering every pulse at its ground-truth PPM-shifted position removes the relative temporal displacement that defines the three classes. Moreover, the required ground-truth center is unavailable during practical inference.

PL achieved 56.520% accuracy, which was higher than AC but substantially lower than NC. PL does not explicitly use the target label; however, locking the window to the local maximum of $\left|r\left[n\right]\right|$ partially compensates for the ±1 μs PPM displacement. The selected maximum may also shift in response to CWI, skywave replicas, delayed-copy overlap, or AWGN. NC avoids this signal-dependent recentering and retains the PPM displacement relative to the same common nominal reference for all three states.

#### 3.4.2. Diagnostic Ablation of Network Architecture and Preprocessing

We next evaluated six architecture and preprocessing variants relative to the full MSST-Net configuration, denoted as A0. A1 removed temporal attention; A2 jointly removed the BiLSTM; A3 removed SE channel recalibration; A4 replaced the multi-scale convolutional block with a single 21-sample kernel; A5 used a lightweight CNN-only architecture; and D1 disabled per-window z-score normalization. All variants used the same training, validation, and test manifest. A2 and A5 modify multiple architectural factors simultaneously and should therefore be interpreted as composite variants rather than single-component ablations (see Figure 15 and Table 8).

The full configuration achieved the highest single-seed values across the reported metrics under the protocol-reference pulse calibration.

From the perspective of temporal modeling, removing the attention mechanism (A1_wo_attention) leads to a performance drop, and further removal of the BiLSTM (A2_wo_bilstm) results in continued degradation. This progressive decline demonstrates that BiLSTM and the attention mechanism jointly form the core of the model’s temporal discriminative capability: BiLSTM captures the contextual dependencies along the time axis within each pulse, while the attention mechanism emphasizes the contribution of the most discriminative temporal regions to the final decision.

At the feature-extraction front end, removing SE channel recalibration (A3_wo_se) or replacing the multi-scale convolution with a single-scale convolution (A4_single_scale) produced small decreases in accuracy. Relative to the full model, A3_wo_se and A4_single_scale decreased in accuracy by 0.08 and 0.21 percentage points, respectively. Because these results were obtained from a single seed, they should be interpreted as descriptive within-protocol differences rather than statistically established effects. The lightweight CNN baseline (A5_cnn_only_lite) achieved markedly lower accuracy than the full model in this single-seed comparison, suggesting that shallow convolutional features alone were insufficient to capture the fine-grained differences among the three PPM states under the tested protocol.

Disabling per-window normalization (D1_wo_sample_norm) caused a severe performance decline, with balanced accuracy approaching the three-class chance level. This result indicates that per-window normalization was important for stabilizing the input dynamic range under the tested conditions. Figure 16 shows the per-class accuracy distribution of each ablation variant. The full model achieves the best overall accuracy and class balance; A1_wo_attention and A2_wo_bilstm show more pronounced degradation in the middle class (On-time), whereas D1_wo_sample_norm exhibits severe class imbalance.

#### 3.4.3. Ablation of the Mixed Ordinal Loss

To isolate the contribution of the physical distance-aware term in Equations (27)–(29), we conducted a paired five-seed ablation comparing conventional cross-entropy (CE) with the proposed mixed ordinal loss $\left(\lambda =0.5\right)$. For each seed (42, 2026, 3407, 7777, and 12,345), the two models used identical initialization, data order, network architecture, preprocessing, data partition, and training settings; thus, the loss function was the only experimental variable. Results are reported as mean ± standard deviation. In addition to ACC, Balanced ACC, and SER, we evaluated the adjacent-error rate for Early ↔ On-time and On-time ↔ Late confusions and the extreme-error rate $E_{ext}$ for direct Early ↔ Late confusions. The latter spans two ordinal levels and therefore most directly reflects the intended effect of the distance-aware term.

As shown in Table 9, the mixed ordinal loss increased the mean ACC from 95.12 ± 0.44% to 95.29 ± 0.31% and the Balanced ACC by 0.16 percentage points, while reducing the SER from  4.88 ± 0.44% to 4.71 ± 0.31%. However, the paired-bootstrap 95% confidence interval for the ACC difference was −0.27 to +0.68 percentage points and included zero; therefore, the results do not support a statistically robust improvement in overall accuracy. The adjacent-error rate decreased from 4.60 ± 0.43% to 4.46 ± 0.30%, corresponding to a relative reduction of 2.96%, but the changes were not consistent across all seeds and the confidence interval also included zero. This reduction should therefore be interpreted as a modest trend.

A more consistent effect was observed for direct Early ↔ Late errors. The extreme-error rate decreased from 0.283 ± 0.021%  to 0.248 ± 0.016%, corresponding to a relative reduction of 12.54%. As shown in Figure 17c, the reduction occurred for all five paired seeds, and the paired-bootstrap 95% confidence interval was −0.051 to −0.021 percentage points. Although the absolute reduction was small because such errors were already rare, its consistent direction supports the intended role of the distance-aware term. Thus, the mixed ordinal loss primarily improves the physical consistency of the predictions by suppressing severe two-level errors rather than substantially increasing overall classification accuracy.

### 3.5. Exploratory Label-Free Domain-Shift Diagnostic on Receiver-Recorded eLoran Waveforms

To evaluate zero-shot simulation-to-real transfer, the frozen seed-42 MSST-Net was applied directly to 1440 candidate pulse windows extracted from archived receiver-recorded eLoran waveforms at three sites, with 480 windows per site. No retraining, fine-tuning, pseudo-labeling, or model-output-based adaptation was performed. Only label-independent site calibration of timing offset, A/B phase-code parity, and waveform polarity was conducted using protocol reference pulses (see Figure 18).

Despite the change from simulated to receiver-recorded waveforms, the model retained stable multi-class behavior without collapsing to a constant output. The Early, On-time, and Late output indices were selected 590, 184, and 666 times, corresponding to pooled shares of 40.97%, 12.78%, and 46.25%, respectively, and an effective class count of 2.68 out of 3. No class accounted for more than half of the predictions at any site. Candidate windows also produced a high mean top-1 confidence of 0.9267 and a low normalized entropy of 0.1711, compared with 0.9210 and 0.1878 for the off-pulse controls. These results show that the frozen model preserved confident, non-degenerate, and broadly site-consistent outputs under direct zero-shot application.

The protocol-reference control yielded an On-time output rate of 13.47%, indicating that the simulated class semantics were not yet fully aligned with the receiver-recorded signals. Therefore, real-waveform demodulation accuracy is not claimed because independent tri-state labels were unavailable. Nevertheless, the observed zero-shot stability identifies MSST-Net as a promising basis for subsequent calibration or domain adaptation using labeled receiver data.

## 4. Discussion

### 4.1. Interpretation of Results

Under the predefined simulation protocol and the group- and frame-disjoint partition, the MSST-Net model trained with Nominal-Center (NC) windows achieved 93.709% accuracy, 93.846% balanced accuracy, and a macro-F1 score of 93.812% on 61,008 held-out simulated pulses. These results show that single-pulse three-state PPM classification is feasible under the modeled composite-interference conditions when all classes share a common nominal timing reference.

The alignment comparison explains why this reference is important. NC retains the class-defining temporal displacement relative to a label-independent center. Actual-Center (AC) alignment instead uses the ground-truth PPM shift to recenter each pulse, removing much of the temporal cue and causing the model to collapse to the On-time class. The signal-driven peak-locking (PL) comparator also partially absorbs the displacement and is sensitive to interference-induced peak shifts. Accordingly, NC achieved 93.709% accuracy, compared with 56.520% for PL and 37.634% for AC.

MSST-Net also outperformed the task-matched lightweight CNN and Linear MSVM baselines on the same test split, and its advantage over Linear MSVM increased as structured interference accumulated. In the separate cross-entropy-controlled comparison, MSST-Net obtained the highest observed accuracy among the four parameter-matched architectures without using an architecture-specific loss. Its higher batch-1 latency, however, indicates an accuracy–efficiency trade-off. Because this architecture comparison used one training seed, it supports only an observed advantage under the evaluated protocol.

### 4.2. Performance Variation Across Interference Types

Performance decreased as structured interference was introduced cumulatively: accuracy declined from 99.725% in Stage 1 to 90.441% in Stage 4. Stage 1 contained AWGN but none of the modeled structured-interference components, whereas Stages 2–4 progressively added CWI, skywave, and XCI. These components can disturb carrier-scale oscillations, broaden or superimpose the pulse envelope, and create temporal overlap between pulses, respectively. The larger separation among the evaluated architectures in the later stages is therefore consistent with their different abilities to represent distorted temporal structure.

These stage-wise differences represent changes in cumulative channel difficulty rather than isolated causal effects of individual interference components. Moreover, the nominal SNR denotes the sequence-level groundwave-to-AWGN power ratio before band-pass filtering and excludes CWI, skywave, and XCI power. Because independently generated SNR–Stage subsets may contain different structured-interference realizations, local non-monotonic changes with nominal SNR should not be interpreted as a pure AWGN response. The single-seed architecture comparison likewise does not establish across-seed statistical significance.

### 4.3. Implications of Ablation Study

The diagnostic ablations indicate that input normalization and temporal modeling contributed most clearly to the observed performance. Disabling per-window normalization reduced balanced accuracy to 33.33%, indicating output collapse under the evaluated setting. Removing temporal attention or jointly removing the BiLSTM produced larger accuracy losses than removing SE recalibration or replacing the multi-scale front end with a single-scale convolution. However, the small differences for the SE and multi-scale variants were obtained from single runs and should not be interpreted as statistically established component effects. The CNN-only variant also changed several architectural factors and therefore represents a composite comparison rather than a single-module ablation.

The five-seed loss ablation provides stronger evidence for the mixed ordinal loss. Relative to conventional cross-entropy, the proposed loss produced only a modest change in overall accuracy but consistently reduced direct Early–Late errors, yielding a 12.54% relative reduction in the extreme-error rate. The distance-aware term should therefore be interpreted primarily as a regularizer that improves the ordinal consistency of residual errors, rather than as the main source of the model’s overall accuracy.

### 4.4. Limitations and Future Work

The quantitative evaluation relies primarily on simulations of AWGN, CWI, skywave propagation, and XCI. The simulator does not fully reproduce receiver nonlinearity and saturation, ADC quantization, oscillator instability, antenna effects, or nonstationary propagation and interference [29]. The skywave and XCI models also omit site- and transmitter-specific effects, including ionospheric dispersion, independent GRI and phase codes, carrier-phase offsets, and waveform variation. The reported metrics therefore apply only to the modeled parameter space. Although the group- and frame-disjoint partition prevents identity leakage across data subsets, it does not provide validation in an independent physical signal domain.

The archived receiver recordings lacked independently verified pulse-level tri-state labels and were used only for the label-free diagnostic in Section 3.5. Their non-collapsed outputs do not establish correct Early/On-time/Late transfer or real-world demodulation accuracy. All supervised metrics in this study were obtained from simulated data, and model predictions were not used as proxy labels. A traceable field-validation protocol will require receiver-recorded or SDR-acquired signals with independently verified pulse-level labels.

The current method classifies pulses independently and does not exploit group-level protocol constraints or coding redundancy. Future work should combine pulse classification with sequence decoding and evaluate nonzero synchronization-reference errors. The three-state distance-aware term could also be extended to richer error costs. Finally, the reported parameter counts and GPU latency do not establish real-time operation, memory use, or energy efficiency on embedded receivers; these properties require hardware-specific evaluation after compression and quantization.

## 5. Conclusions

This study investigates eLoran single-pulse three-state PPM classification under a predefined controlled composite-interference simulation protocol. A unified received-signal model covering groundwave propagation, skywave multipath, continuous-wave interference, cross-station interference, and Gaussian noise formalizes single-pulse state recognition as a temporally constrained classification task. The main contributions are: (1) a label-independent Nominal-Center sampling strategy that retains the class-defining ±1 μs displacement relative to a common nominal timing reference and avoids target-dependent actual-center alignment; (2) an MSST-Net multi-scale spatiotemporal network that combines multi-receptive-field convolutions, SE channel recalibration, BiLSTM temporal modeling, and attention-based aggregation to represent microsecond-scale shifts, local carrier oscillations, and pulse-envelope distortions; and (3) a mixed ordinal loss with physical distance awareness that embeds the ordinal topology of the three PPM states to suppress cross-level misclassification.

Under the predefined composite-interference simulation pipeline and the group- and frame-disjoint partition, the seed-42 model achieved 93.71% accuracy, 93.85% balanced accuracy, and 93.81% macro-F1 on 61,008 held-out channel-realized test windows generated from 372 test pulse identities belonging to 62 test pulse groups across 164 SNR–Stage configurations. The corresponding SER was 6.29%, comprising 5.88% adjacent-state errors and 0.41% direct Early–Late errors. Accuracy decreased from 99.72% in Stage 1 to 90.44% in Stage 4 as interference components were introduced cumulatively. On the same split, NC achieved 93.709% accuracy, compared with 56.520% for the implemented peak-lock comparator and 37.634% for the oracle AC diagnostic. MSST-Net also exceeded the task-matched lightweight CNN and Linear MSVM baselines, which achieved 87.462% and 86.605%, respectively. In a separate five-seed paired experiment, the mixed ordinal loss reduced the extreme Early–Late error rate by 12.54%.

In the parameter-matched modern-architecture benchmark, MSST-Net (CE) achieved 92.557% ACC, compared with 91.229% for the Transformer, 89.336% for CNN-BiLSTM, and 88.293% for ResNet1D. It also achieved the highest observed accuracy in each stage, although its batch-1 GPU latency was higher than those of the external baselines. These single-seed results support an accuracy advantage under the evaluated protocol, not across-seed statistical significance or unconditional efficiency superiority.

These findings support the proposed Nominal-Center and multi-scale spatiotemporal modeling framework under the controlled simulation assumptions adopted in this study. They do not establish equivalent performance in operational eLoran receivers or national PNT deployments. In particular, the primary dataset uses $\varepsilon_{sync,g}=0$, and robustness to nonzero synchronization-reference errors has not been established. The receiver-recorded analysis is diagnostic only and does not provide real-waveform demodulation accuracy. Future work will require traceable receiver-recorded or SDR-acquired datasets with independently verified pulse-level tri-state labels, together with explicit synchronization-error evaluation.

## Figures and Tables

**Figure 1:**
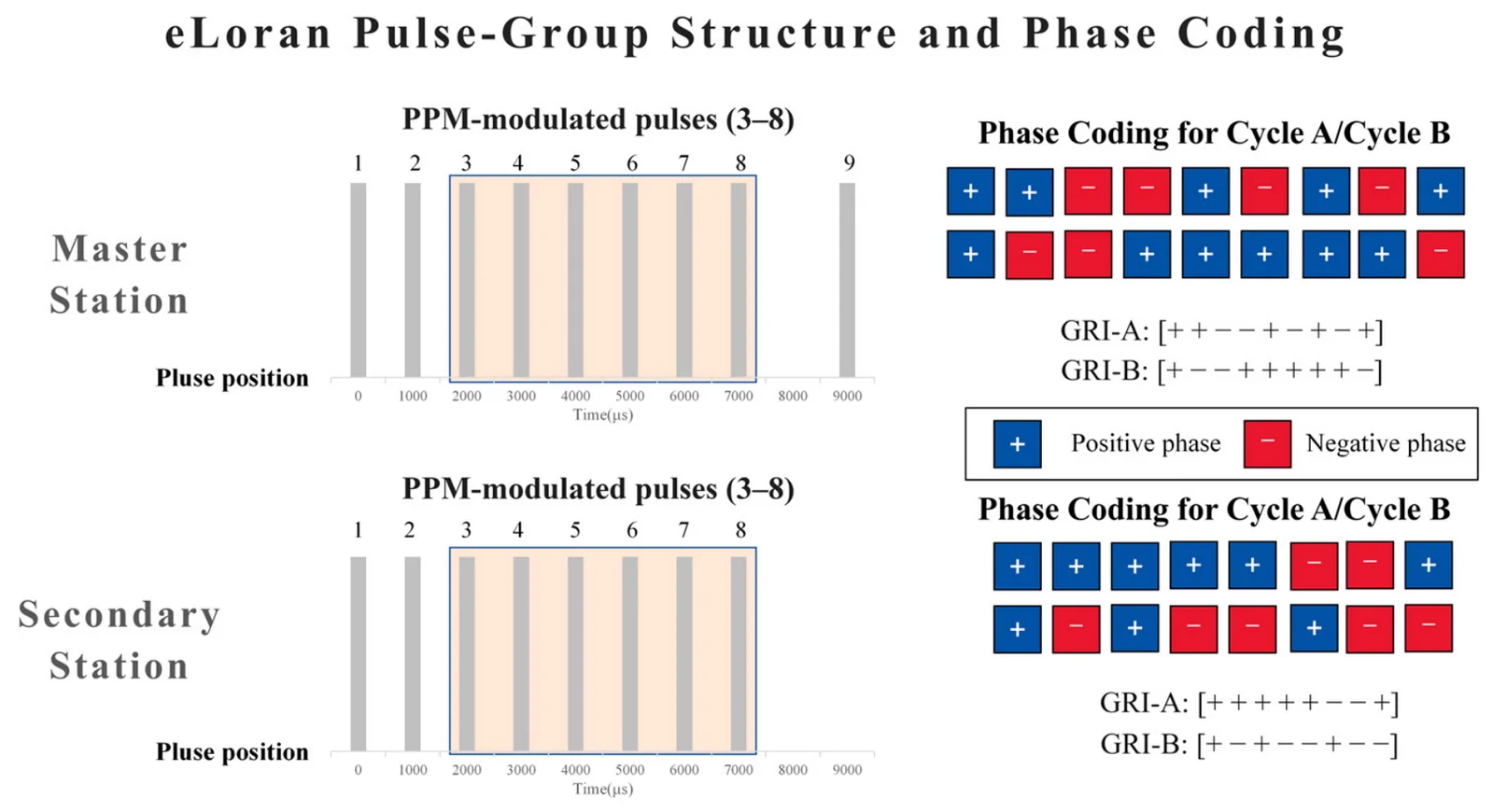
Structure of eLoran master and secondary pulse groups with A/B phase coding across successive pulse groups.

**Figure 2:**
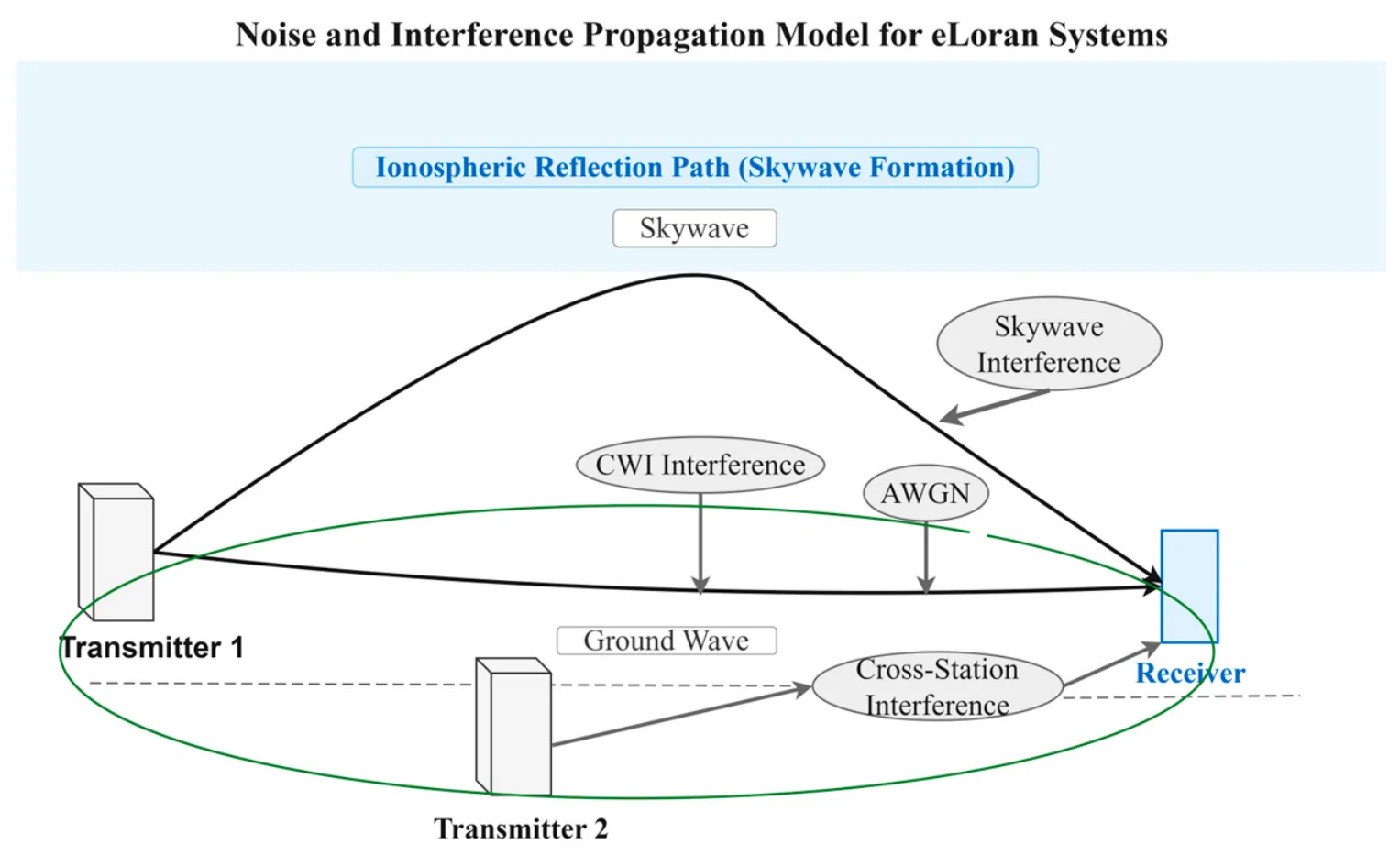
Noise and interference propagation model for eLoran systems.

**Figure 3:**
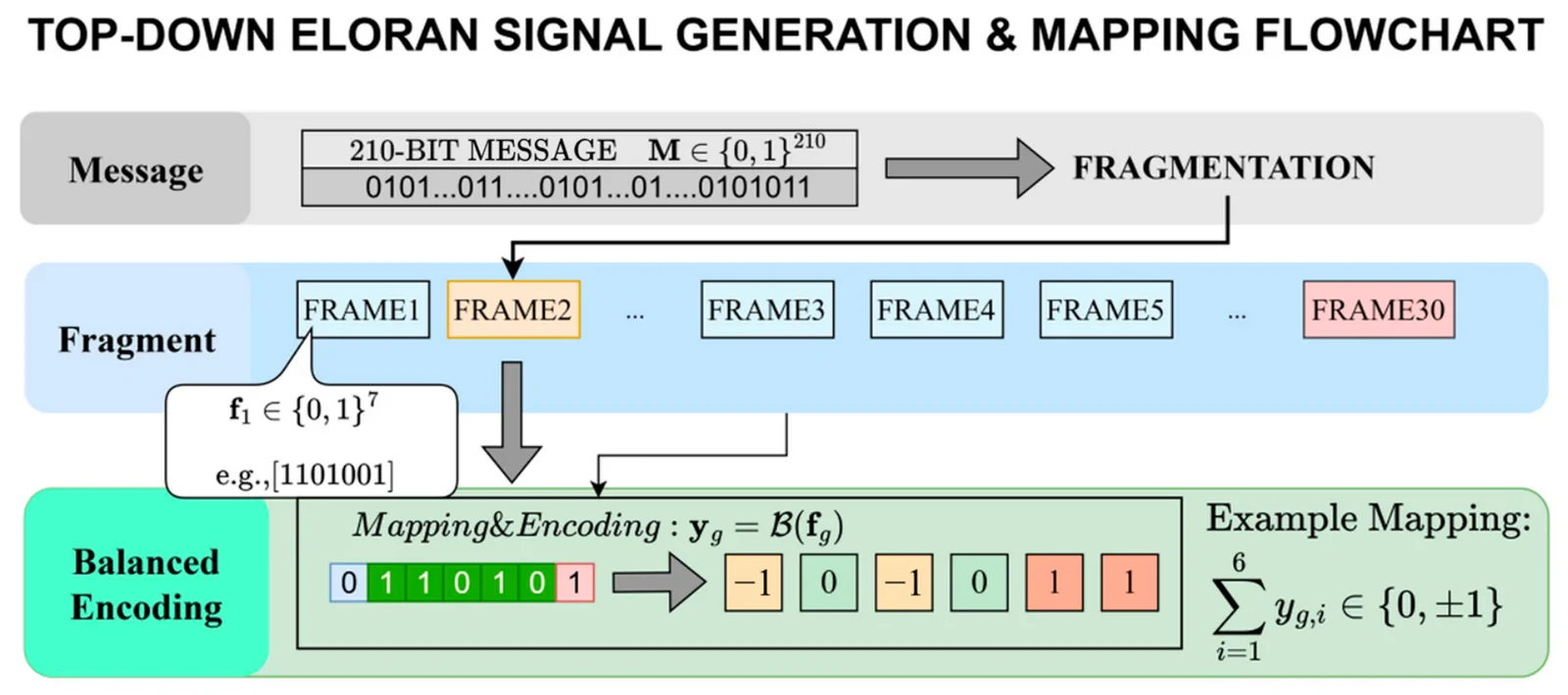
Protocol-driven data generation pipeline.

**Figure 4:**
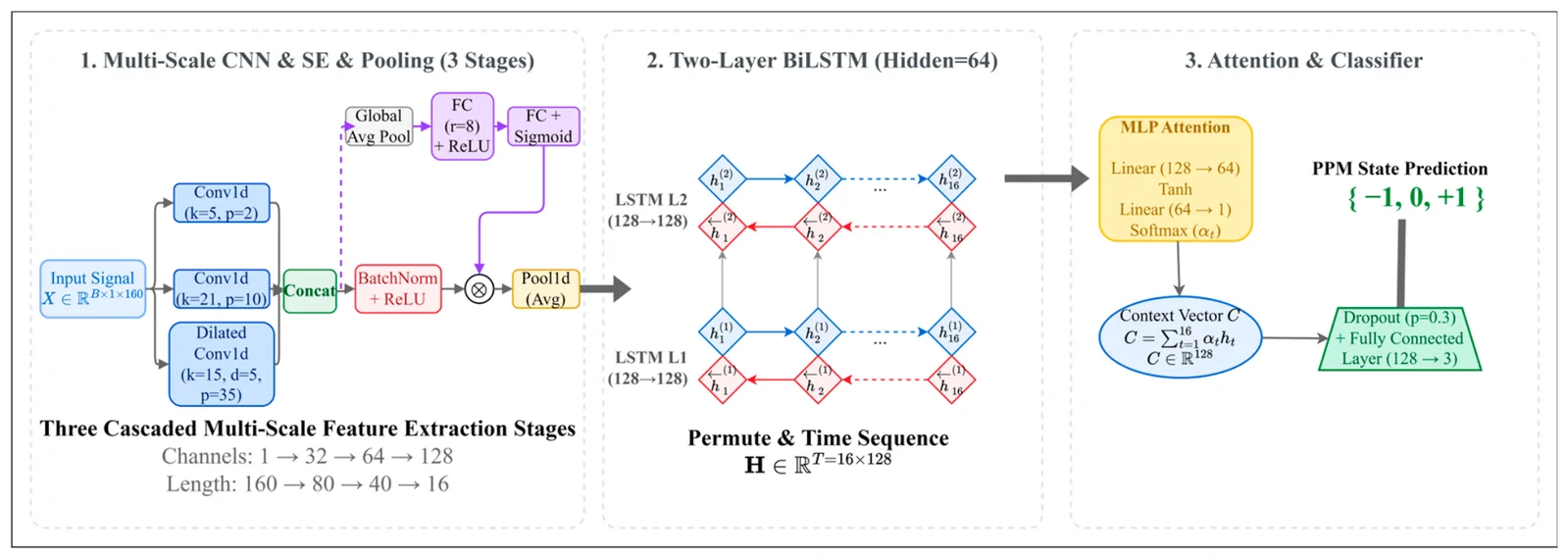
Overall architecture of MSST-Net.

**Figure 5:**
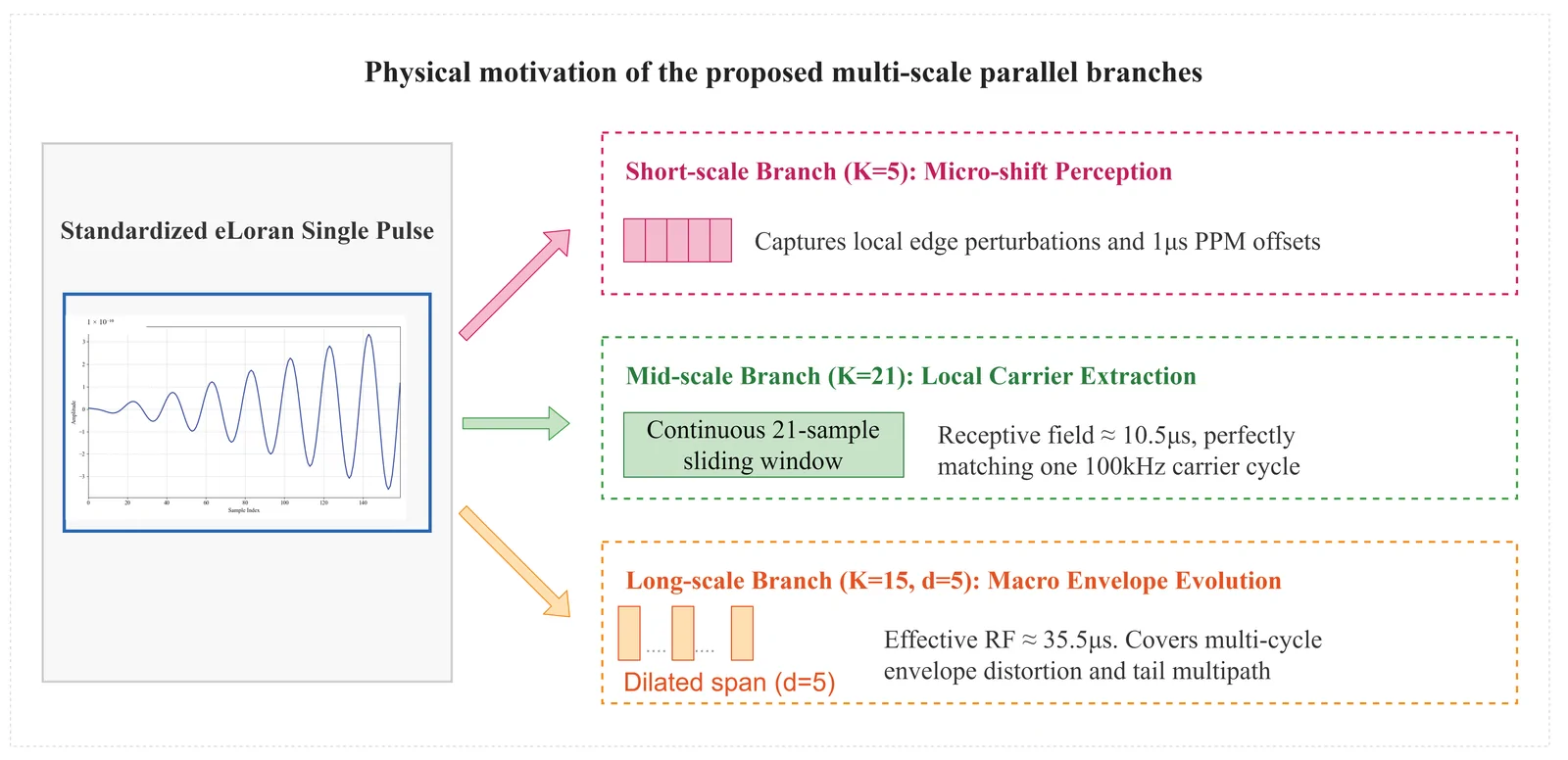
Multi-scale feature extraction and the mapping between receptive fields and eLoran pulse physical characteristics.

**Figure 6:**
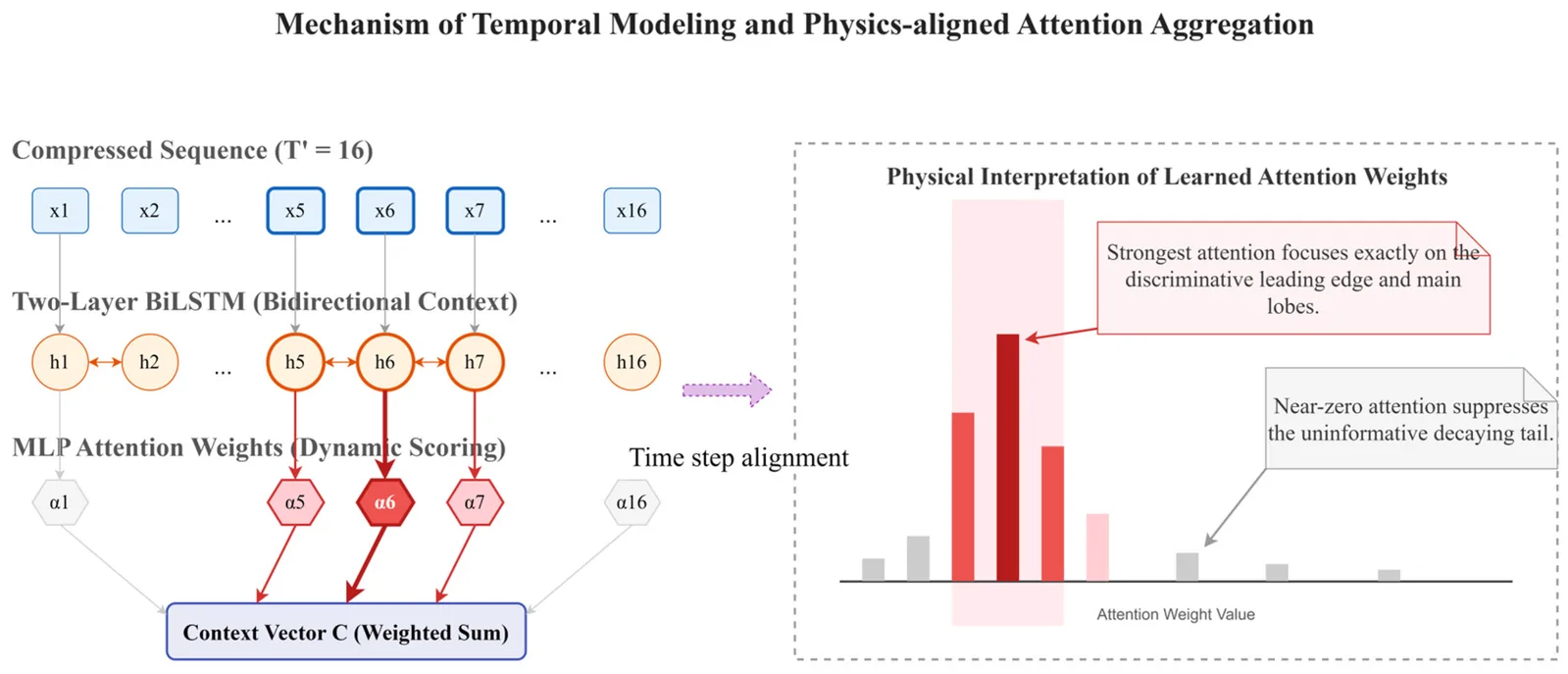
BiLSTM attention visualization: blue inputs, orange hidden states, red high weights, and gray low weights; high weights mark the pulse leading edge.

**Figure 7:**
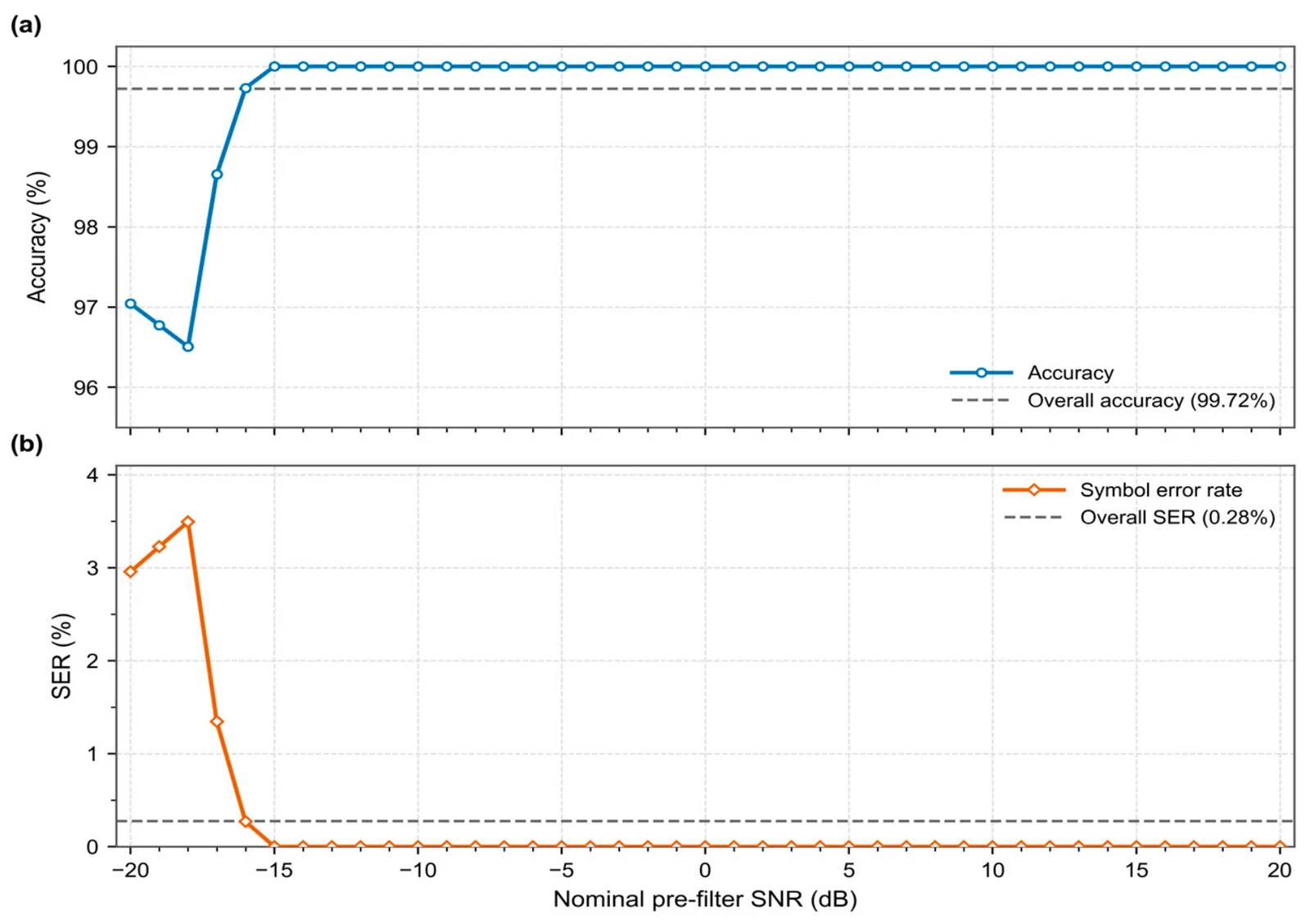
AWGN-only performance: (**a**) ACC versus nominal SNR; (**b**) SER versus nominal SNR.

**Figure 8:**
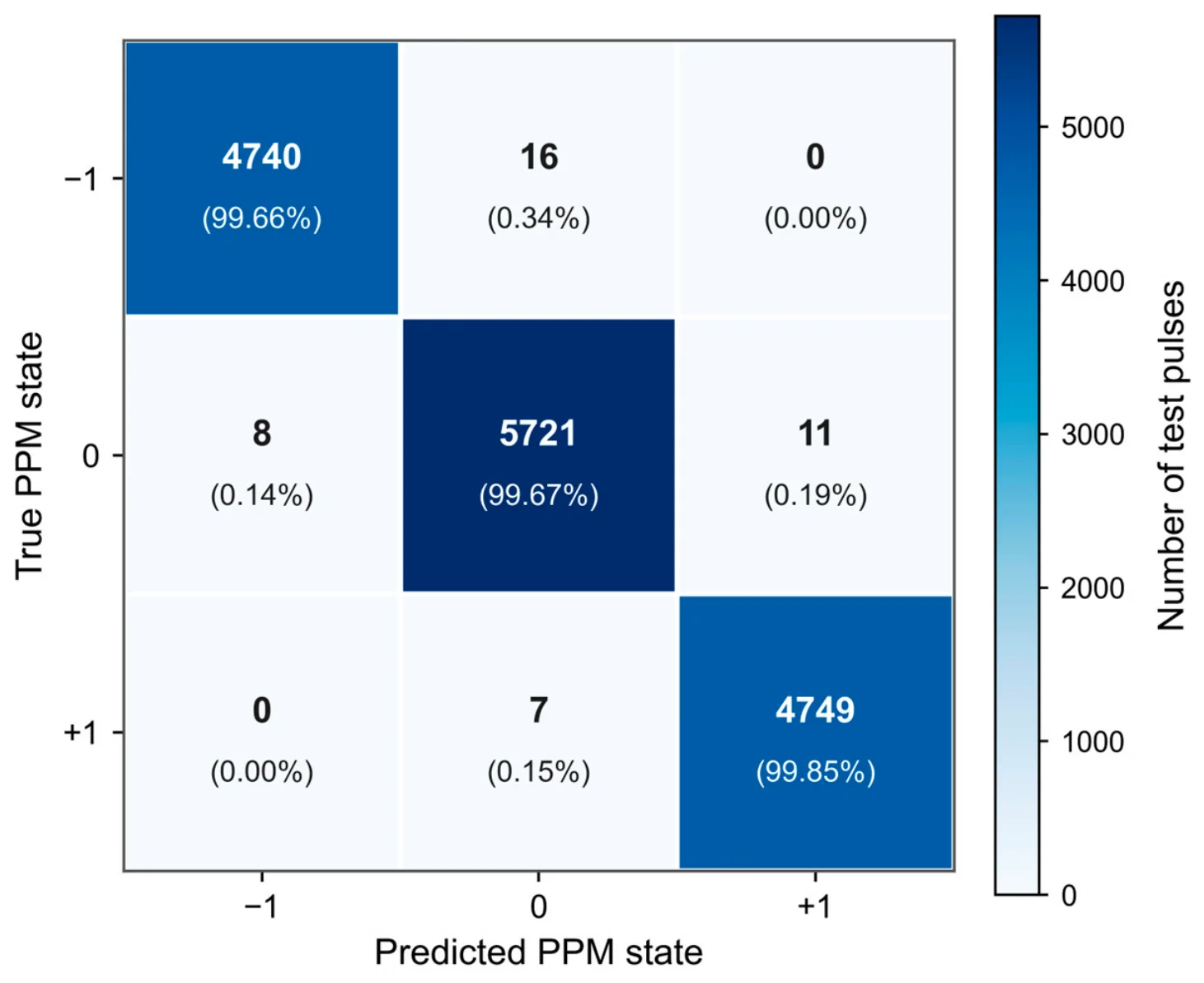
Confusion matrix of MSST-Net on the AWGN-only dataset.

**Figure 9:**
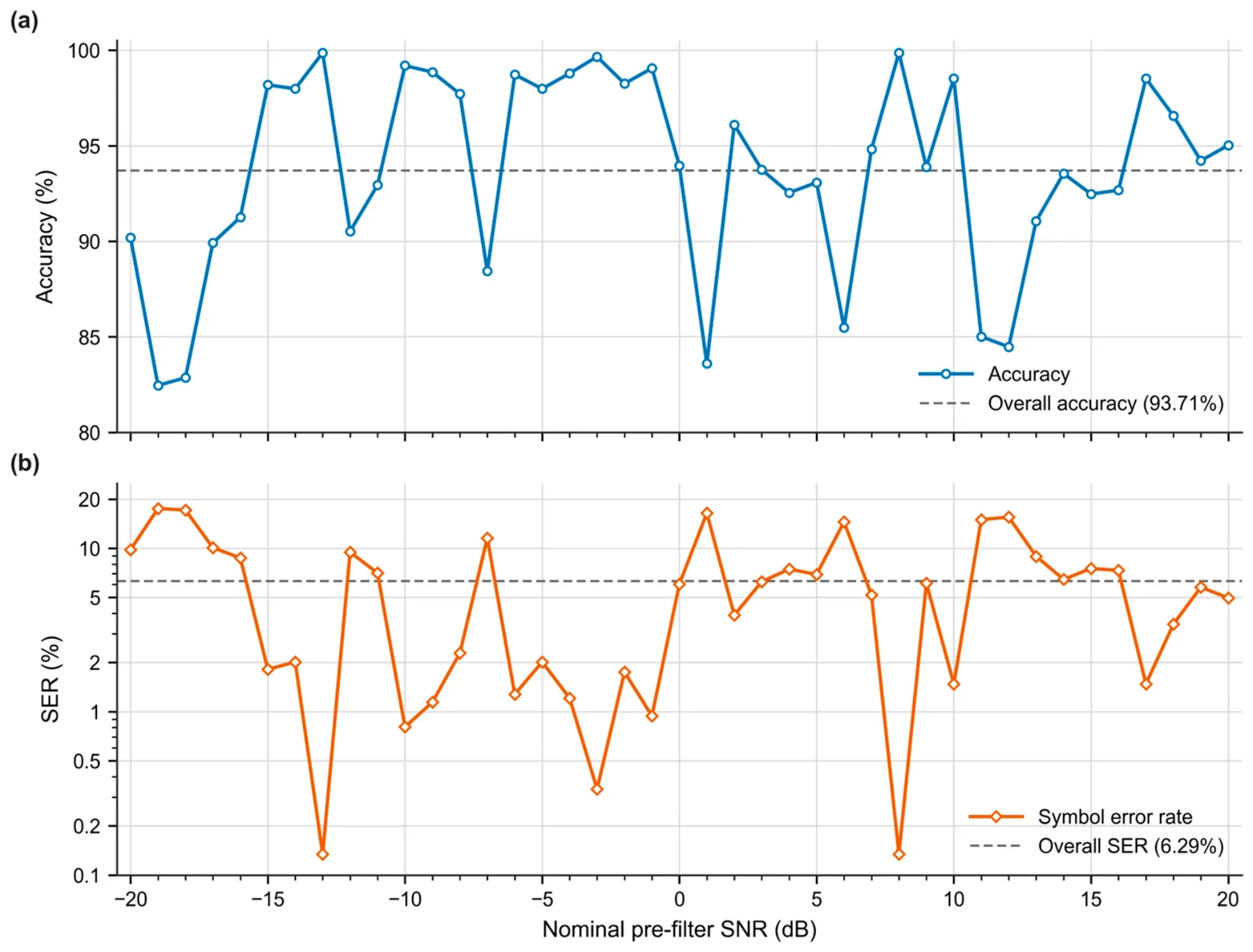
Mixed-interference performance: (**a**) ACC versus nominal SNR; (**b**) SER versus nominal SNR.

**Figure 10:**
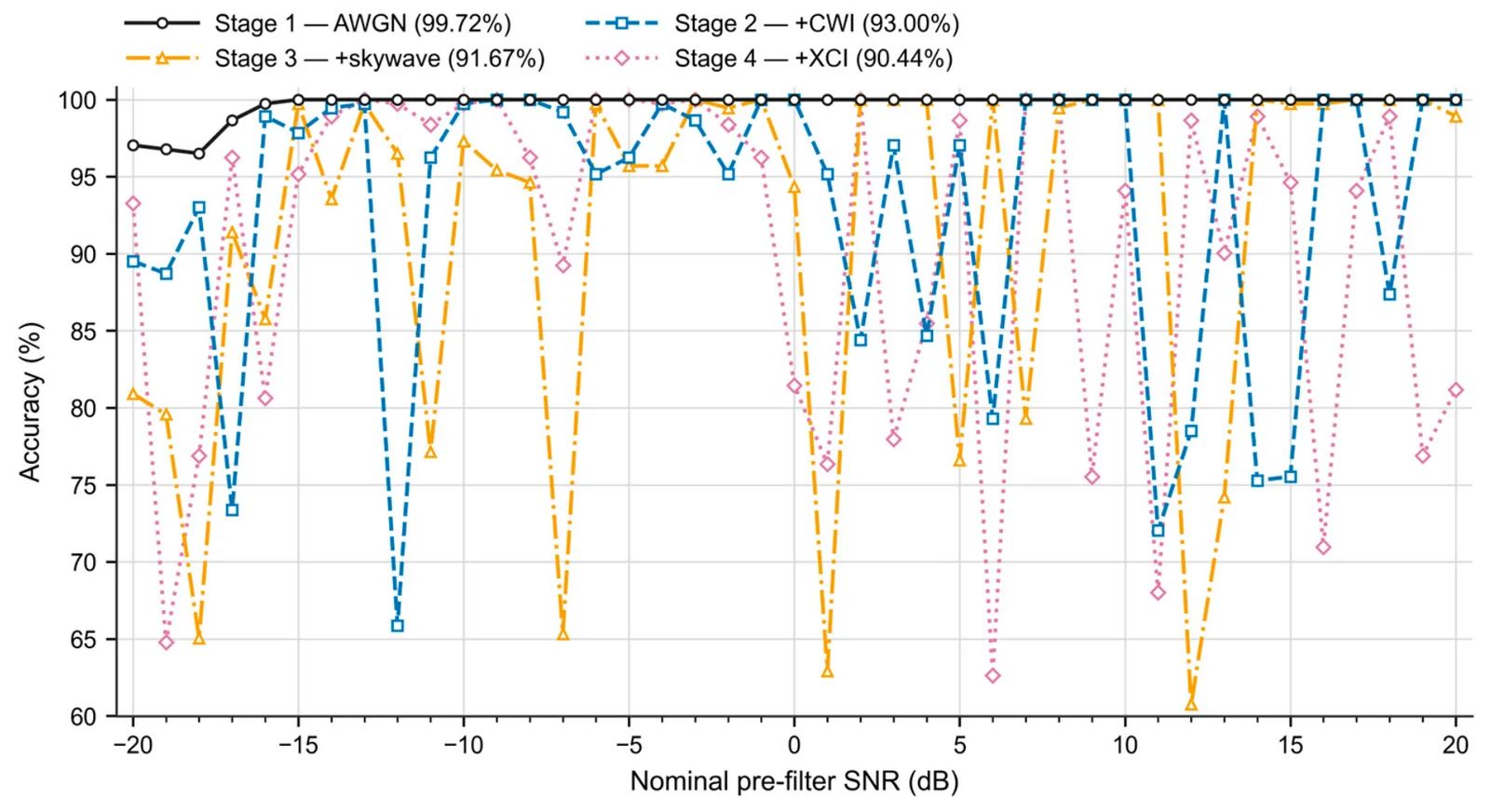
ACC versus nominal SNR for the four cumulative interference stages in the mixed-interference dataset.

**Figure 11:**
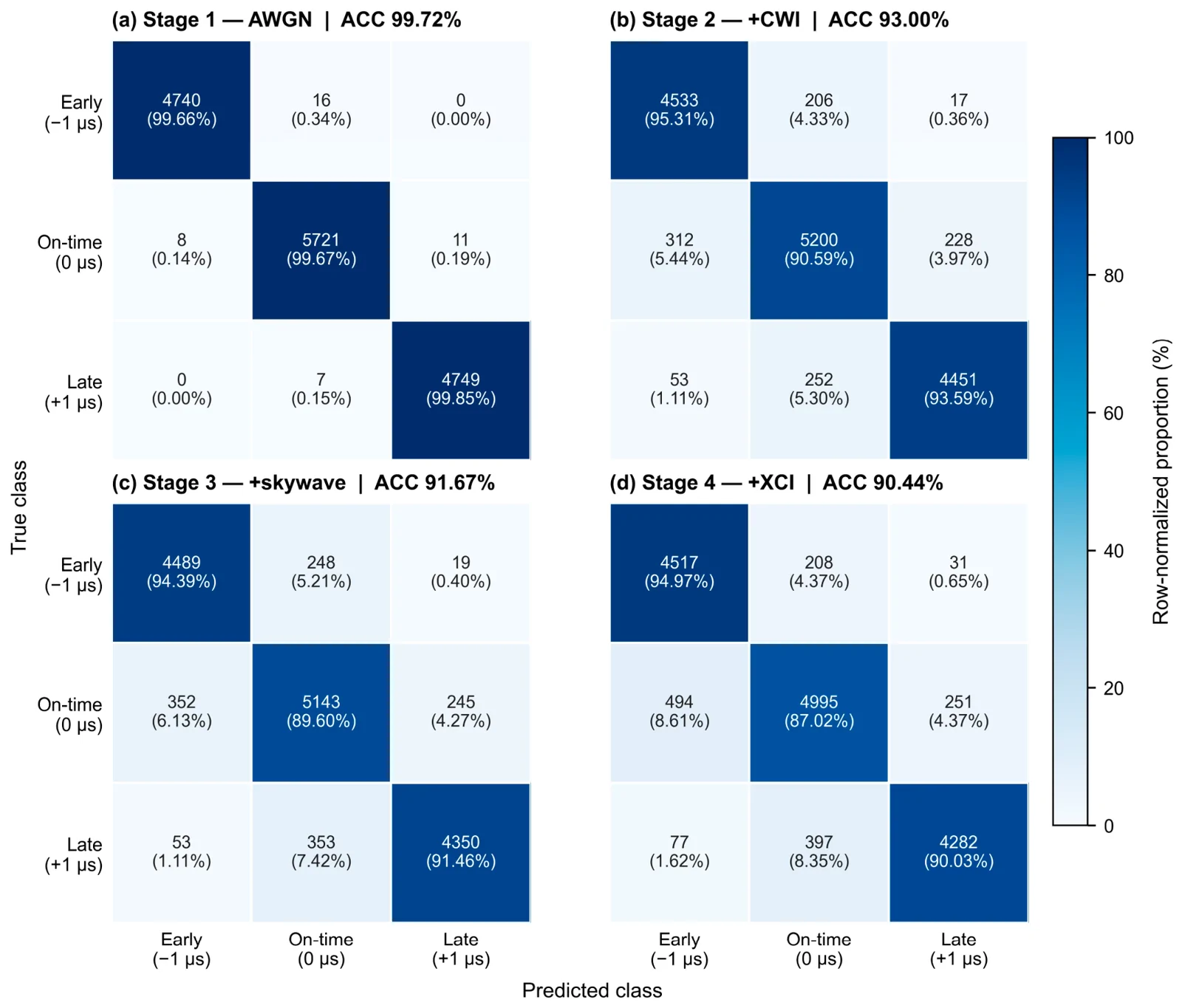
Confusion matrix of MSST-Net on the mixed-interference dataset.

**Figure 12:**
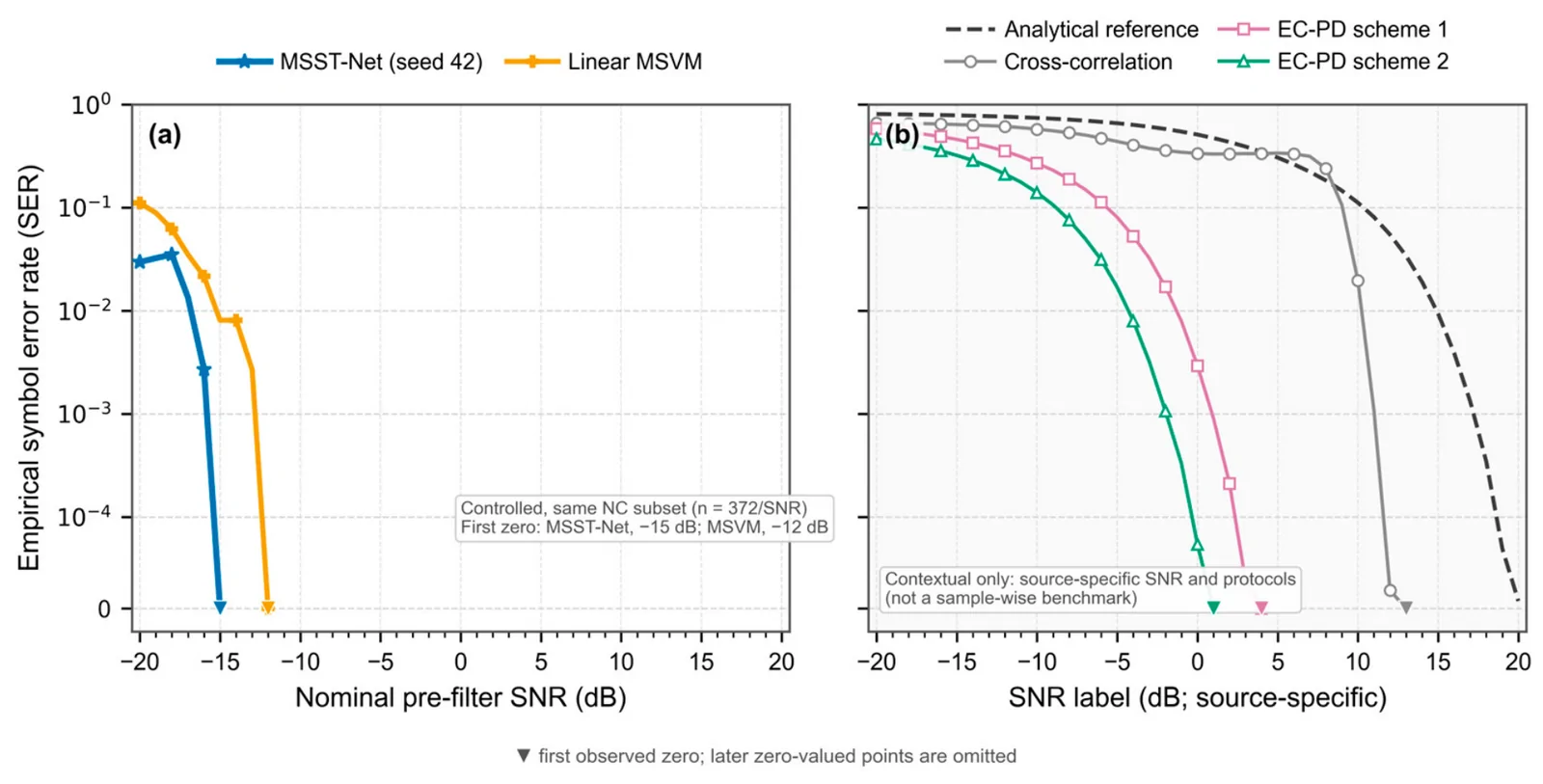
SER–SNR comparisons from −20 to 20 dB: (**a**) MSST-Net versus linear MSVM under the same Stage 1 Nominal-Center protocol; (**b**) MSST-Net versus analytical and conventional DSP references under their respective AWGN formulations. Panel (**b**) is contextual and is not a sample-wise comparison with panel (**a**).

**Figure 13:**
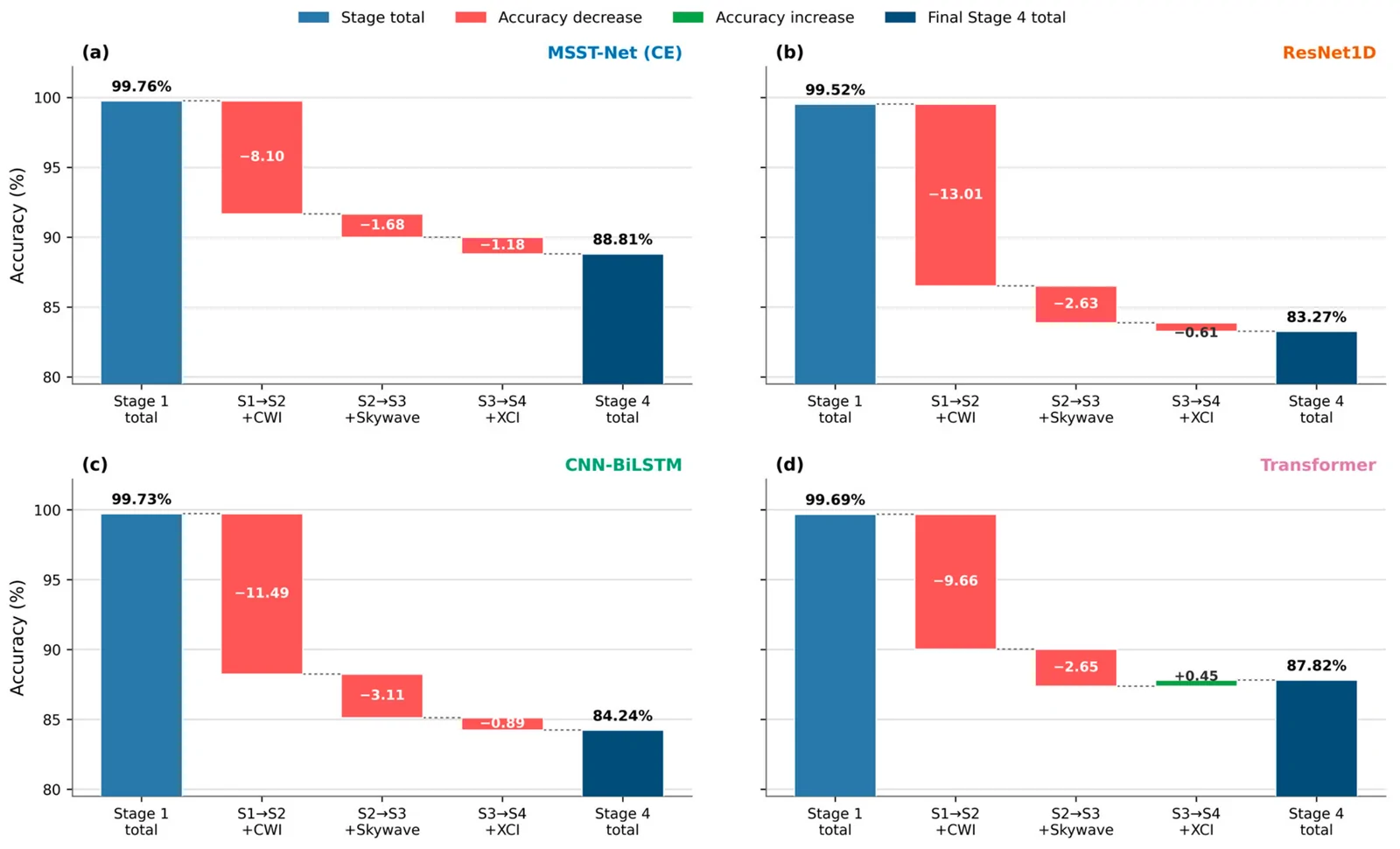
Stage-wise accuracy of (**a**) MSST-Net (CE), (**b**) ResNet1D, (**c**) CNN-BiLSTM, and (**d**) Transformer on the common pooled test set. The first and last bars show the absolute Stage 1 and Stage 4 accuracies; the floating bars show the differences between successive stage subsets. Red and green indicate decreases and increases, respectively. These differences are descriptive and do not isolate the causal effect of an individual interference component.

**Figure 14:**
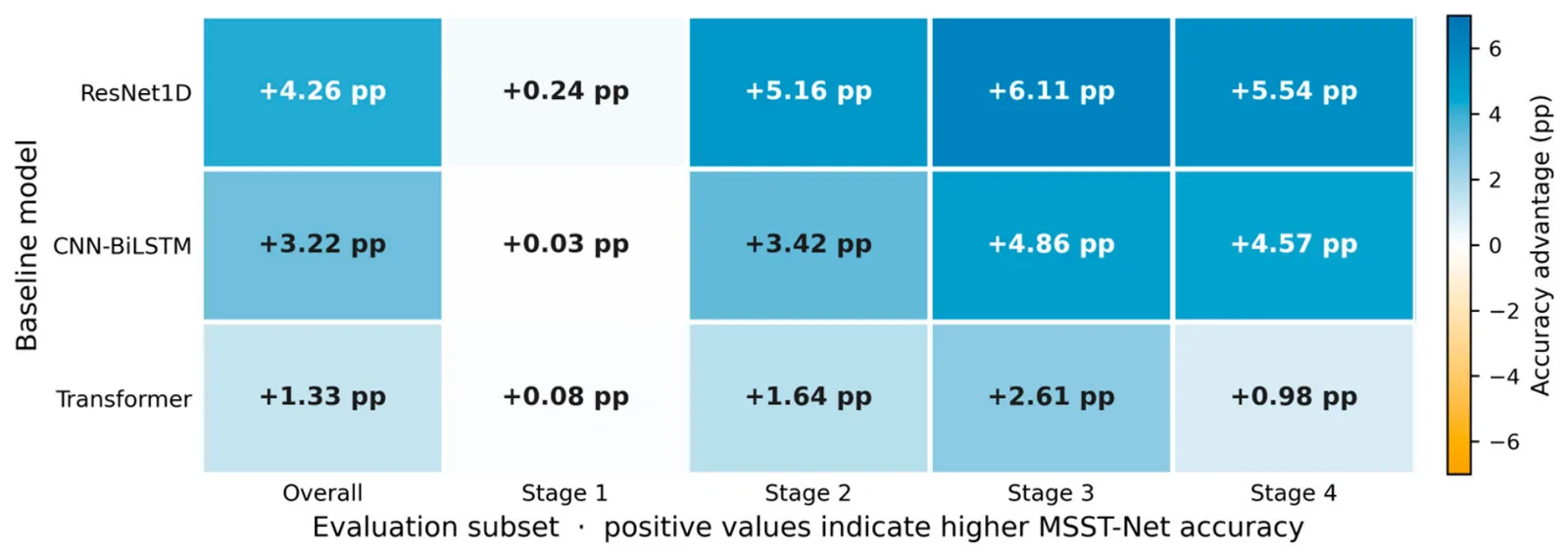
Accuracy advantage of MSST-Net (CE) over each external baseline. Values are absolute percentage-point differences; positive values favor MSST-Net. The figure shows single-seed observations, not across-seed confidence intervals or statistical significance.

**Figure 15:**
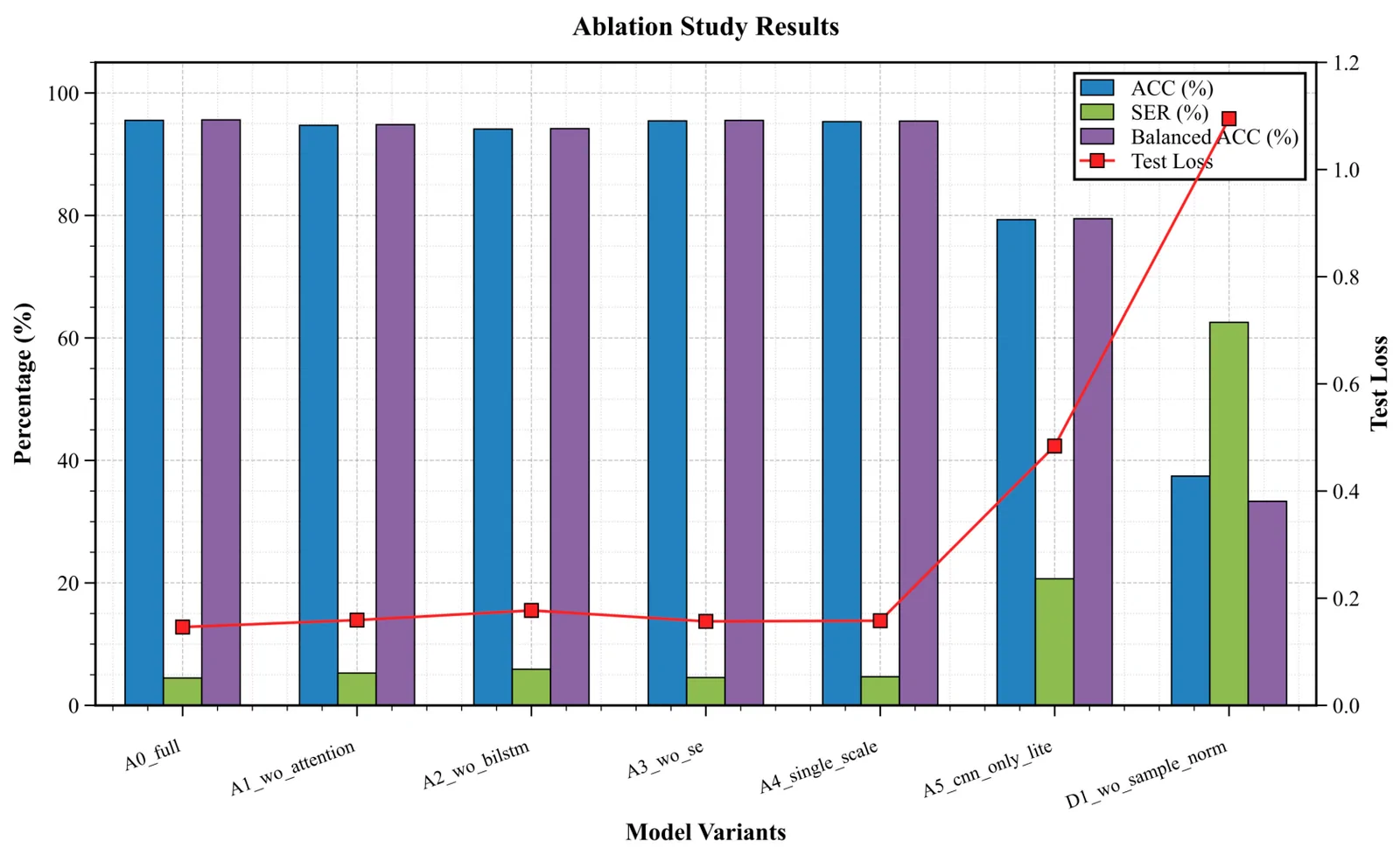
Comprehensive performance comparison of the proposed model and its ablated variants in terms of accuracy, balanced accuracy, and symbol error rate.

**Figure 16:**
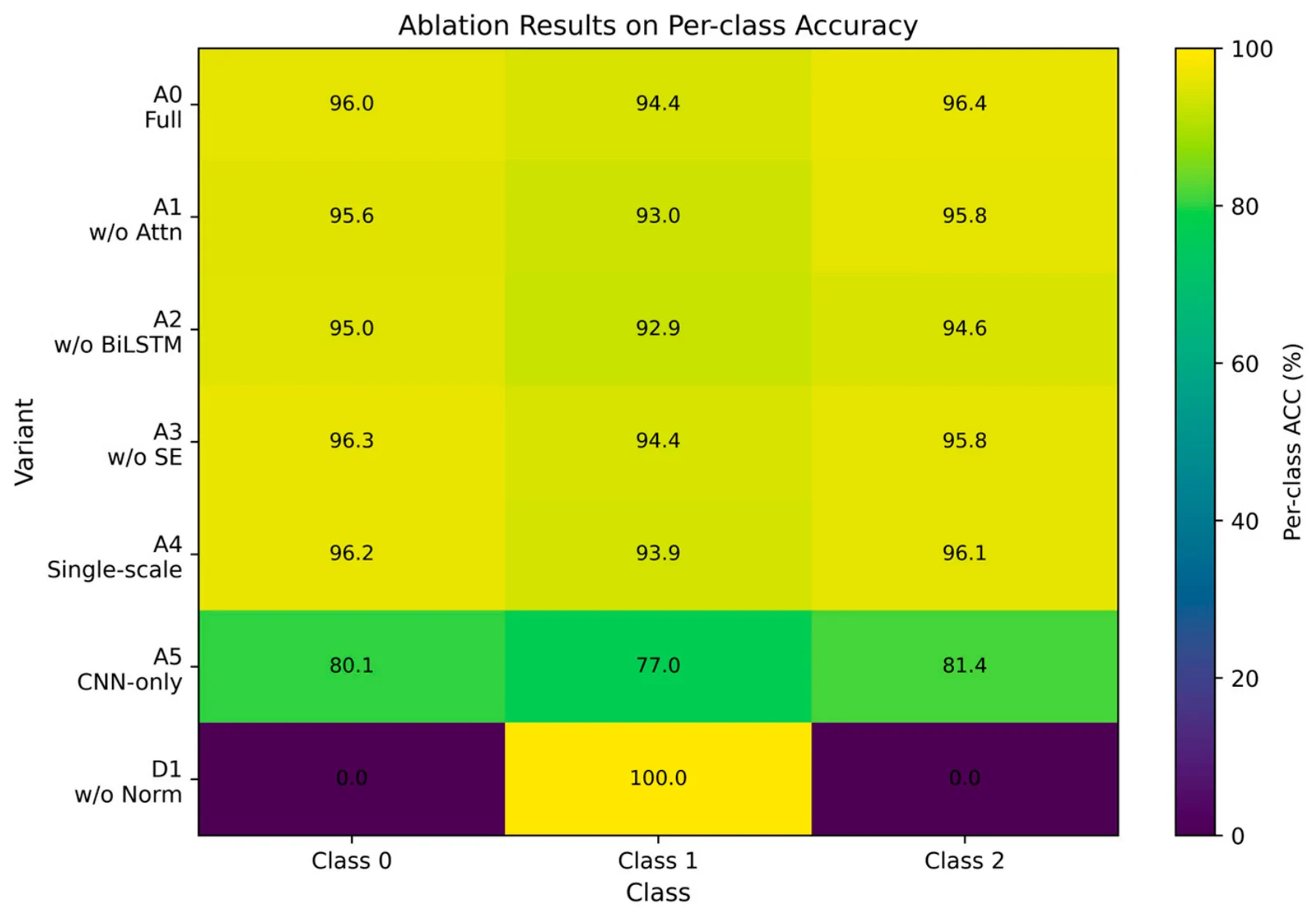
Per-class accuracy heatmap for different ablation variants.

**Figure 17:**
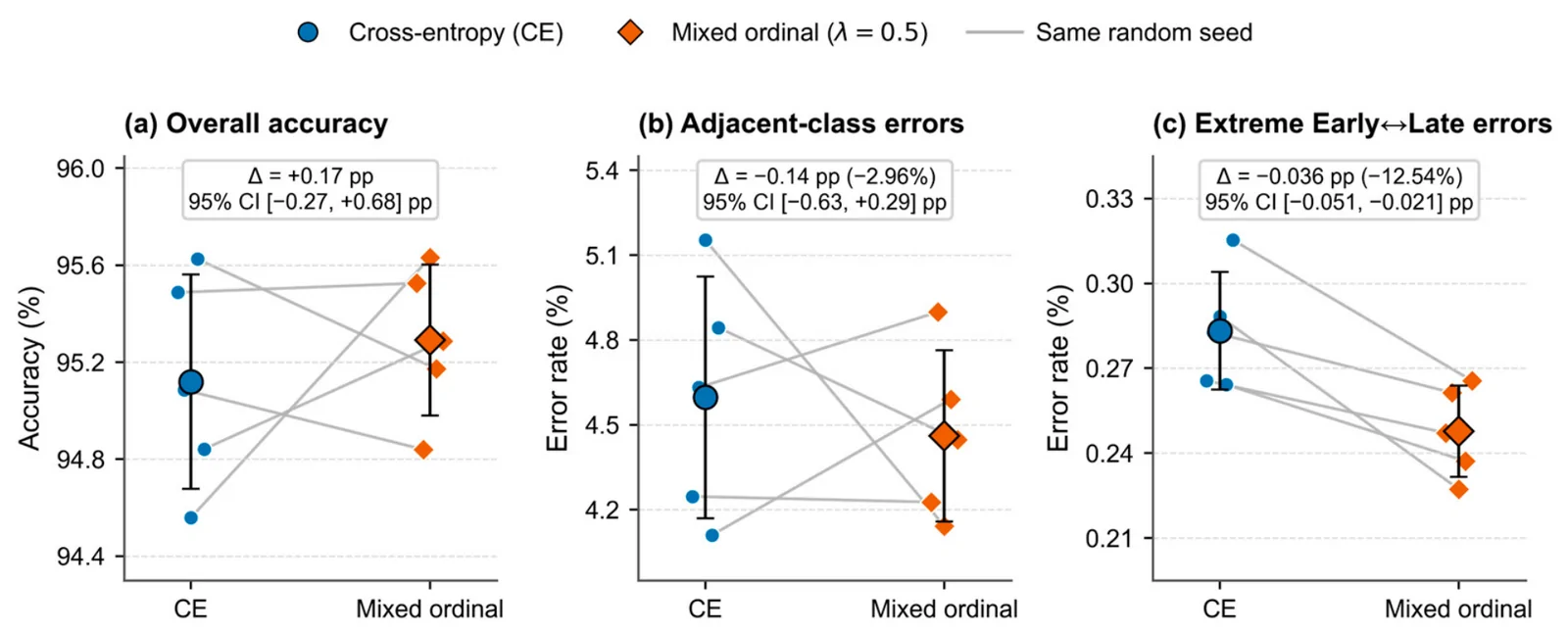
Paired five-seed comparison of cross-entropy and the proposed mixed ordinal loss $\left(\lambda =0.5\right)$: (**a**) Overall classification accuracy; (**b**) adjacent-class error rate, including Early ↔ On-time and On-time ↔ Late confusions; and (**c**) extreme Early ↔ Late error rate. Thin gray lines connect the results obtained using the same random seed. Large markers and error bars denote the across-seed mean and one standard deviation, respectively. The annotations report the mean paired difference and its bootstrap 95% confidence interval.

**Figure 18:**
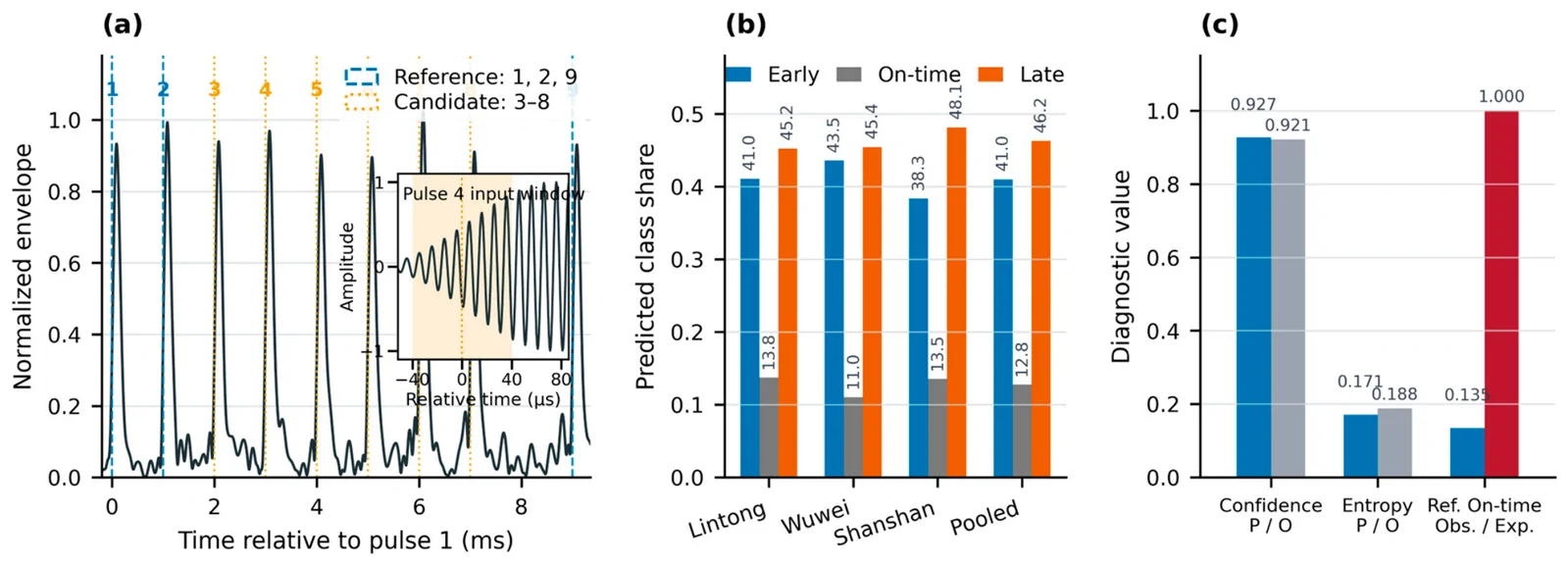
Zero-shot analysis of receiver-recorded eLoran waveforms. (**a**) Representative band-pass-filtered pulse group from Wuwei; the inset shows the local waveform context, with the shaded region marking the 160-sample Nominal-Center input window. (**b**) Predicted-class distributions at the three sites and for the pooled dataset. (**c**) Confidence and normalized-entropy diagnostics for candidate and off-pulse windows, together with the protocol-reference On-time control. Independent pulse-level tri-state labels were unavailable; therefore, the results characterize zero-shot output behavior rather than real-waveform demodulation accuracy.

**Table 1:** Cumulative interference stages and corresponding channel components.

Stage	AWGN	CWI	Skywave	XCI
1	√	×	×	×
2	√	√	×	×
3	√	√	√	×
4	√	√	√	√

Note: The channel components are added cumulatively from Stage 1 to Stage 4.

**Table 2:** Experimental structure and evaluation matrix for Section 3.

Subsection	Research Objective	Test Dataset	Key Analysis Dimension	Primary Metrics
Section 3.1	Validate baseline anti-noise capability	AWGN-only (−20 to 20 dB)	Performance knee point and convergence characteristics in low-SNR regimes	ACC, SER
Section 3.2	Evaluate robustness under compound interference	Mixed interference (Stage 1–4)	Interference coupling effects and phase-dependent performance variations	ACC, SER, macro-F1
Section 3.3	Compare with task-matched traditional and contemporary DL baselines	Common pooled Stage 1–4 test set; source-specific contextual curves	Architecture competitiveness, stage-wise behavior, capacity, and latency	ACC, Balanced ACC, macro-F1, SER, parameters, latency
Section 3.4	Quantify architectural component contributions	Mixed interference test set	Within-protocol performance differences and ordinal error structure	ACC, Balanced ACC, Loss
Section 3.5	Examine zero-shot behavior on receiver-recorded waveforms	Three archived 1 MHz receiver recordings	Output non-collapse and simulation-to-real domain mismatch	Predicted-class shares, effective class count, confidence, entropy

**Table 3:** MSST-Net performance on the AWGN-only dataset.

SNR (dB)	Accuracy (%)	SER (%)
0	100.00	0.00
−8	100.00	0.00
−12	100.00	0.00
−14	100.00	0.00
−16	99.73	0.27
−18	96.51	3.49
−20	97.04	2.96

**Table 4:** MSST-Net performance on the mixed-interference dataset.

SNR (dB)	Accuracy (%)	SER (%)	Balanced Accuracy (%)
−20	90.19	9.81	90.32
−16	91.26	8.74	91.46
−12	90.52	9.48	90.81
−8	97.72	2.28	97.74
−4	98.79	1.21	98.81
0	93.95	6.05	94.27
4	92.54	7.46	92.58
8	99.87	0.13	99.87
12	84.48	15.52	84.70
16	92.67	7.33	92.79
20	95.03	4.97	95.18

**Table 5:** Stage-wise performance on the predefined simulated test split.

Stage	Test Samples	Average Accuracy (%)	Balanced Accuracy (%)	SER (%)
1	15,252	99.72	99.73	0.28
2	15,252	93.00	93.16	7.00
3	15,252	91.67	91.82	8.33
4	15,252	90.44	90.68	9.56

**Table 6:** Task-matched comparison of contemporary deep-learning architectures under the common CE-based protocol.

Model	Parameters	Accuracy (%)	Balanced Accuracy (%)	Macro-F1 (%)	SER (%)	Batch-1 Latency (ms)
MSST-Net (CE)	353,432	92.557	92.759	92.683	7.443	2.289
ResNet1D	359,035	88.293	88.378	88.516	11.707	1.833
CNN-BiLSTM	354,596	89.336	89.489	89.524	10.664	1.078
Transformer	350,787	91.229	91.426	91.368	8.771	1.612

Note: All models used the same 160-sample Nominal-Center input, group and frame-disjoint partition strategy, training configuration, and evaluation protocol described in Section 2.5.3. The reported results are single-run estimates obtained with the same random seed.

**Table 7:** Ablation results for different sample-construction strategies.

Sampling Strategy	Center Determination	Accuracy (%)	SER (%)	Macro-F1 (%)
NC	Nominal timing reference	93.709	6.291	93.812
AC	Ground-truth actual center	37.634	62.366	18.229
PL	Signal-driven center estimate	56.520	43.480	53.272

**Table 8:** Ablation study results for MSST-Net components.

Variant	Accuracy (%)	SER (%)	Balanced Accuracy (%)	Test Loss
A0_full	95.53	4.47	95.61	0.1462
A1_wo_attention	94.72	5.28	94.83	0.1592
A2_wo_bilstm	94.10	5.90	94.18	0.1772
A3_wo_se	95.45	4.55	95.52	0.1568
A4_single_scale	95.32	4.68	95.41	0.1580
A5_cnn_only_lite	79.32	20.68	79.48	0.4840
D1_wo_sample_norm	37.45	62.55	33.33	1.0949

**Table 9:** Five-seed paired ablation of cross-entropy and the mixed ordinal loss on the mixed-interference test set.

Loss Function	Accuracy (%)	SER (%)	Balanced Accuracy (%)	Adjacent Error (%)	Extreme Error (%)
Cross-Entropy	95.12 ± 0.44	4.88 ± 0.44	95.22 ± 0.44	4.60 ± 0.43	0.283 ± 0.021
Mixed Ordinal, λ=0.5	95.29 ± 0.31	4.71 ± 0.31	95.38 ± 0.31	4.46 ± 0.30	0.248 ± 0.016
Difference	+0.17 pp	−0.17 pp	+0.16 pp	−0.14 pp	−0.035 pp
Relative change	+0.18%	−3.52%	+0.17%	−2.96%	−12.54%

Note: Results are reported as mean ± standard deviation over five paired random seeds. “pp” denotes percentage points. Adjacent errors include Early ↔ On-time and On-time ↔ Late confusions; extreme errors include direct Early ↔ Late confusions. Lower values are better for SER and both error-rate metrics.

## Data Availability

The data that support the findings of this study are available from the corresponding author upon reasonable request.
